# Modulation of Estrogen Receptor Activity by the Phytoalexin Tuberosin Produced from Elicited Kudzu (*Pueraria lobata*)

**DOI:** 10.1021/acs.jnatprod.5c00192

**Published:** 2025-06-20

**Authors:** Jorge A. Belgodere, Jack R. Elliott, Megan C. Benz, G. Wills Kpeli, Steven Elliott, Isaac J. Ponder, Geoffroy E. R. Sanga Pema, Peng Ma, Sophie R. Dietrich, Thomas Cheng, Khoa Nguyen, Syreeta L. Tilghman, Binghao Zou, Muralidharan Anbalagan, Brian G. Rowan, Robert H. Newman, Mark Mondrinos, Jayalakshmi Sridhar, Thomas E. Wiese, Simak Ali, Van T. Hoang, Bridgette M. Collins-Burow, Elizabeth C. Martin, Hamed K. Abbas, Stephen M. Boué, Matthew E. Burow

**Affiliations:** † Tulane Department of Medicine, Section of Hematology & Medical Oncology, Tulane University Health Science Center, New Orleans, Louisiana 70112, United States; ‡ Department of Biological and Agricultural Engineering, Louisiana State University and Agricultural Center, Baton Rouge, Louisiana 70803, United States; § Tulane Cancer Center, 5783 Tulane University, New Orleans, Louisiana 70112, United States; ∥ Department of Biomedical Engineering, Tulane University, New Orleans, Louisiana 70112, United States; ⊥ Xavier University School of Pharmacy, Xavier University, New Orleans, Louisiana 70125, United States; # Pharmaceutical Sciences Division, College of Pharmacy and Pharmaceutical Sciences, Florida A&M University, Tallahassee, Florida 32307, United States; 7 Department of Structural and Cellular Biology, 12255 Tulane University School of Medicine, New Orleans, Louisiana 70112, United States; 8 Department of Biology, North Carolina A&T State University, Greensboro, North Carolina 27411, United States; 9 Department of Chemistry, Xavier University of Louisiana, New Orleans, Louisiana 70125, United States; 10 Department of Surgery and Cancer, Imperial College London Hammersmith Hospital Campus, W12 0NN London, U.K.; 11 U.S. Department of Agriculture, Agricultural Research Service, Southeast Area, Stoneville, Mississippi 38776, United States; 12 U.S. Department of Agriculture, Agricultural Research Service, Southern Regional Research Center, New Orleans, Louisiana 70179, United States

## Abstract

Kudzu’s invasive nature has contributed to its classification as a weed, as it frequently outcompetes native plant species, leading to extensive overgrowth. Efforts to control kudzu have proven challenging, with moderate success using physical or biological agents. In this study, we evaluated the effects of two such control agents, ultraviolet C radiation and *Myrothecium verrucaria*, to significantly increase the production of tuberosin, a phytoalexin isoflavone. Our findings demonstrate that estrogenic activity of tuberosin is cell-type-dependent, displaying antagonist or competitive inhibition when combined with 17-β-estradiol in the estrogen receptor (ER) positive cell lines MCF-7 and T-47D, while showing dose-dependent agonist activity in HEK293 cells transfected to express both ER receptors (α and β). Tuberosin was shown to modulate ER pathways, alter ER-mediated gene expression, and increase cell proliferation in a dose-dependent manner while maintaining expression of the ERα protein. Binding affinity and docking simulations confirmed tuberosin binding to the ERα pocket in a similar but weaker manner compared to synthetic estrogen. Tuberosin-treated endothelial cells suppressed vascular network assembly and maturation without affecting the cellular proliferative capacity. The presented studies leverage current kudzu management methods to naturally produce tuberosin, examine cell-type-specific effects, and support further investigation as an antiestrogen for breast cancer treatment.

## Graphical abstract

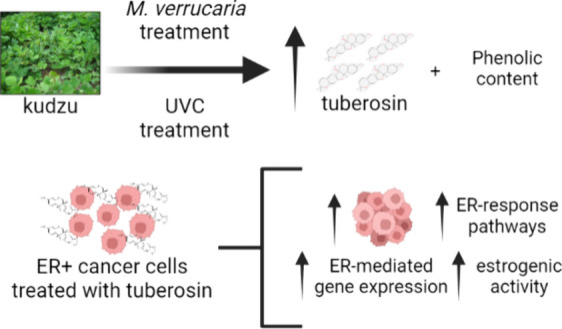

Kudzu (*Pueraria lobata)* is a perennial leguminous vine native to East Asia and was initially introduced to the United States to prevent soil erosion. However, due to kudzu’s rapid growth rate, high leaf area indices, elevated photosynthetic rates, and frequent rooting at stem nodes, it quickly became an aggressive competitor to native trees and shrubs.^1^ Kudzu vines grow up to 18 m in a season (up to 30 cm/day),^1^ causing damage to forest ecosystems and infrastructure (∼$1.5 million/year^2^) and leading to increased economic and environmental burden.^1,3^ Due to these issues, kudzu was removed from the approved plant list in 1953 and was officially labeled a weed in 1970.^3^

Various methods have been developed to control kudzu growth, including aerial or ground-based herbicide applications. However, their high costs and substantial use restrictions have led to alternative approaches, e.g., highly virulent bioherbicides.^2–4^ The fungal pathogen *Myrothecium verrucaria* was identified as a potential bioherbicide, but results were inconsistent, suggesting that combining herbicides with fungal bioherbicides might offer more effective control.^4,5^ Physical agents, like ultraviolet C (UVC) irradiation, have been reported to elicit flavone production in rice leaves,^6^ carrots,^7^ and other vegetable leaves^8^ through self-defense mechanisms.

Beyond its invasive status, kudzu offers potential health benefits by predominately containing several flavonoids in its leaves and roots, which have been used in traditional medicine in English and Chinese literature to treat fever, dysentery, diarrhea, diabetes, and cardiovascular disease.^9,10^ One of the key isoflavones, puerarin, has demonstrated efficacy in cardiovascular disease treatment across various studies.^10^ Other isoflavones in kudzu, such as daidzein, genistein, and formononetin, are present as either aglycones or glycosides and characterized as phytoestrogens.^11^ Phytoestrogens interact with estrogen receptors (ERs), exhibiting estrogenic and antiestrogenic activities. In humans, two types of estrogen receptors, ERα and ERβ, are distributed across distinct tissues. Phytoestrogens display relatively weak estrogenic activity, approximately 100–1,000 times lower than 17-β-estradiol (E2), depending on the specific ER subtype they interact with. However, phytoestrogens may reach plasma concentrations 100 times higher than endogenous estrogens,^12,13^ which may partially explain their antiestrogenic activity through competitive inhibition of endogenous E2.^13,14^ Phytoestrogen dual activity has led to increased interest in identifying new plant sources and methods to enhance phytoestrogen production, particularly isoflavones.

Environmental factors, including germination conditions, stress, or elicitor treatments, can alter isoflavone concentrations in kudzu and other legumes. For example, soybean plants produce glyceollin I, II, and III—phytoalexins derived from daidzein—in response to fungal stress.^15^ We previously examined the antiestrogenic activity of soy glyceollin in vitro^15^ and in vivo,^16^ demonstrating these compounds, unlike constitutive soy isoflavones such as daidzein and genistein, exert significant antiestrogenic effects on ER signaling. This was further correlated to reduced estrogen-induced proliferation in breast cancer cells. Additionally, we have shown fungal elicitation of green and snow pea resulted in significantly increased (+)-pisatin (another isoflavone) concentrations leading to context-dependent estrogen activity.^17^

Tuberosin, a pterocarpan primarily found in kudzu root, was previously shown to be produced through *Pueraria tuberosa*, a species from the same genus.^18^ Given its chemical structure, production of tuberosin likely follows a similar pathway to that of other elicited legume-based plants.^15^ This highlights kudzu’s potential as a functional food, though its safety, biological characterization, and elicitation methods need to be established before human consumption can be recommended. Despite growing interest, limited data are currently available on the effects of elicitor-treated kudzu and tuberosin. This study aims to characterize the biological effects of kudzu-derived tuberosin. Our specific objectives were as follows: (1) demonstrate and quantify tuberosin production from elicited kudzu leaves, (2) assess estrogenic and antiestrogenic effects of tuberosin in MCF-7 and T-47D breast cancer cell lines, (3) evaluate receptor-specific activity for ERα and ERβ using HEK293 cells, (4) determine ERα binding affinity for tuberosin compared to E2, and (5) examine the effects of tuberosin treatment on ER-mediated biological processes, including gene expression (RNA sequencing and PCR), cell proliferation (Alamar Blue), and ERα protein levels (JESS analysis).

## Results and Discussion

### UVC and *M. verrucaria* Elicitation of Kudzu Leaves Significantly Increases Tuberosin Production

*Pueraria lobata* (kudzu) was previously researched for potential biopharmaceutical applications, particularly estrogenic activity.^19^ Prior studies have investigated elicited crude extracts effects on mice,^20^ fractionalized extracts,^19^ and in combination with other extracts.^21^ Here, we sought to elicit and identify key phytoalexins that may contribute to their reported bioactivity. Nonelicited and elicited kudzu leaves were evaluated using UPLC-PDA. Tuberosin was identified with a characteristic peak approximately at 7 min and was present in nonelicited kudzu (black spectrum, Figure 1a). The production of tuberosin was elevated in kudzu elicited by UVC (>0.1 abundance, red spectrum, Figure 1a) and *M. verrucaria* (<0.1 abundance, green spectrum, Figure 1a). Two other flavonoids were identified: robinin at 4.8 min was found in both control and UVC-elicited kudzu samples,^22^ and daidzein at 5.6 min was found at elevated levels after *M. verrucaria* elicitation. Research by Aisayh et al. with soybean seedlings^23^ has shown that daidzein levels increased after treatment with another fungus, *Rhizopus oryzae.* We further quantified tuberosin concentration in kudzu leaves (Figure 1b) and determined that tuberosin levels in UVC-treated samples (245 ± 1.3 μg/g) were ∼2-fold higher (*p* < 0.0001) compared to those of *M. verrucaria*-elicited samples (123 ± 1.7 μg/g). These results present an alternative source for tuberosin, with UVC-elicitation proving the most effective. These results support UVC treatment or similar approaches to stress the plant not only to facilitate removal but also to valorize this abundant resource through tuberosin production, thus offering an effective strategy to manage invasive species such as kudzu. Based on these findings, we next sought to evaluate the estrogenic effects of tuberosin.

**Figure 1:**
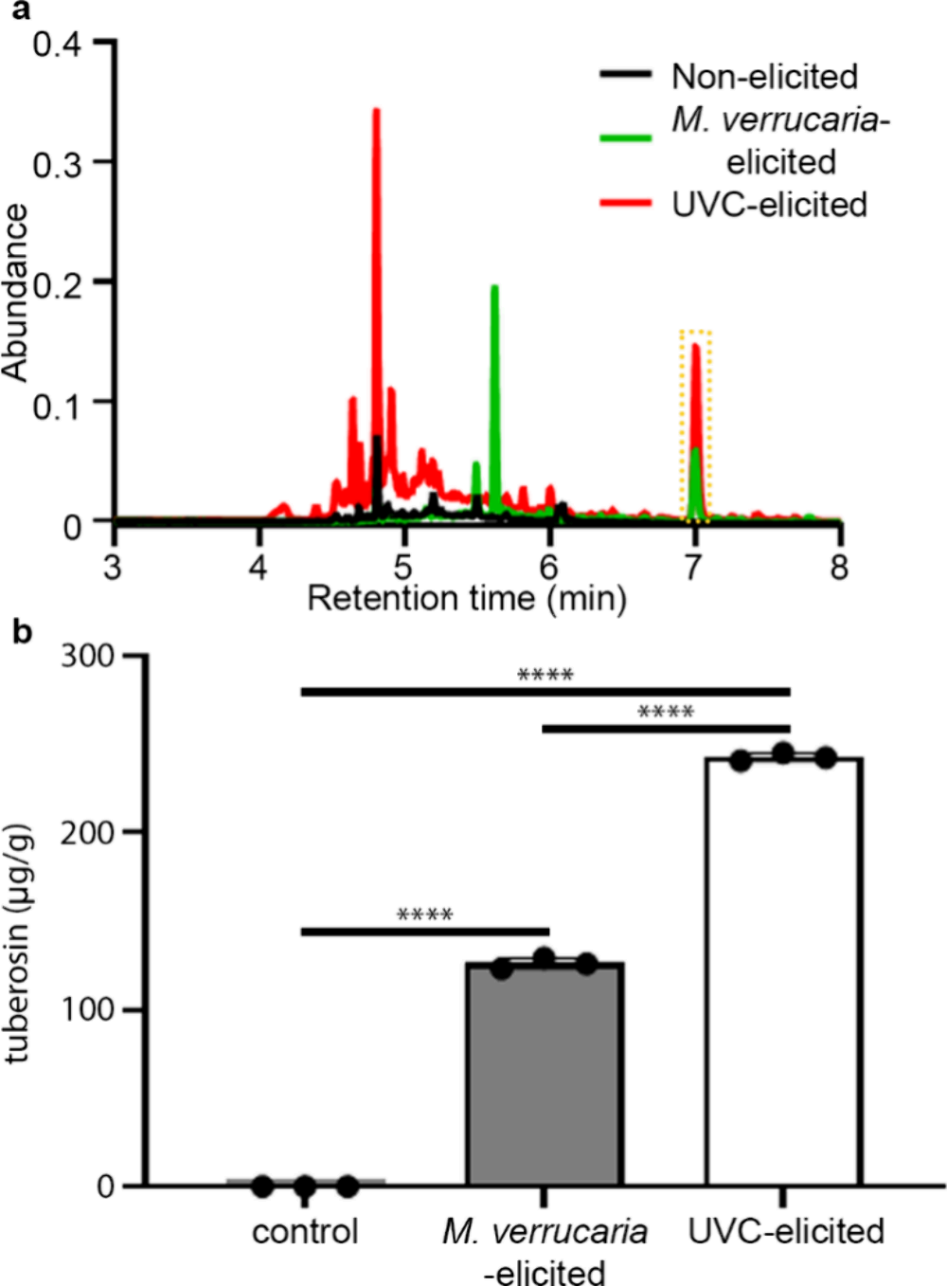
(a) UPLC-PDA chromatograms (TIC) of the MeOH extracts of nonelicited (black), *M. verrucaria*-elicited (green), and UVC-elicited (red) kudzu. Tuberosin was identified at ∼7 min retention time and highlighted in the dashed yellow box. (b) Kudzu leaves initially contained no detectable tuberosin, but after UVC or *M. verrucaria* elicitation there is a significant increase. Data represent mean ± SEM of 3 independent experiments. PDA wavelength at 285 nm. One-way ANOVA, Dunnett’s multiple comparisons test. ****p < 0.0001.

### Tuberosin Modulates Estrogenic Activity in Cells with Exogenous and Endogenous ER Expression

While kudzu was previously investigated in other biological models, the estrogenic activity of tuberosin has not been determined. Therefore, we sought to evaluate changes in estrogen signaling induced by treatment with purified/isolated tuberosin in cells that transiently express ERs (ERα and ERβ) and ER-positive (ER+) breast cancer cell lines (MCF-7 and T-47D).^22^ In HEK293 cells transiently transfected with ERα (HEK293-ERα) and treated with tuberosin (1 μM and 10 μM), ERα activity significantly increased (*p* < 0.0001) in a dose-dependent manner compared to the DMSO control (Figure 2a). Estrogenic activity induced by E2 was 4.2-fold higher than that induced by tuberosin treatment (10 μM) in HEK293-ERα cells (Figure 2a), while ERβ activity was not significantly altered by treatment with tuberosin (Figure 2a). To determine the antagonistic effects of tuberosin, HEK293-ER cells were treated with E2 and tuberosin in combination. Compared to E2-treated cells, treatment with tuberosin (10 μM) significantly suppressed E2-induced ERα and ERβ activity by 2.8-fold (*p* < 0.001) and 3.75-fold (*p* < 0.0001), respectively, in HEK293-ER cells (Figure 2b). Despite these antagonistic effects, estrogenic activity was significantly higher in cells treated with combined tuberosin (10 μM) and E2 compared to DMSO control conditions (*p* < 0.05), indicating partial inhibition of ERα and ERβ activity by tuberosin in this context.

**Figure 2:**
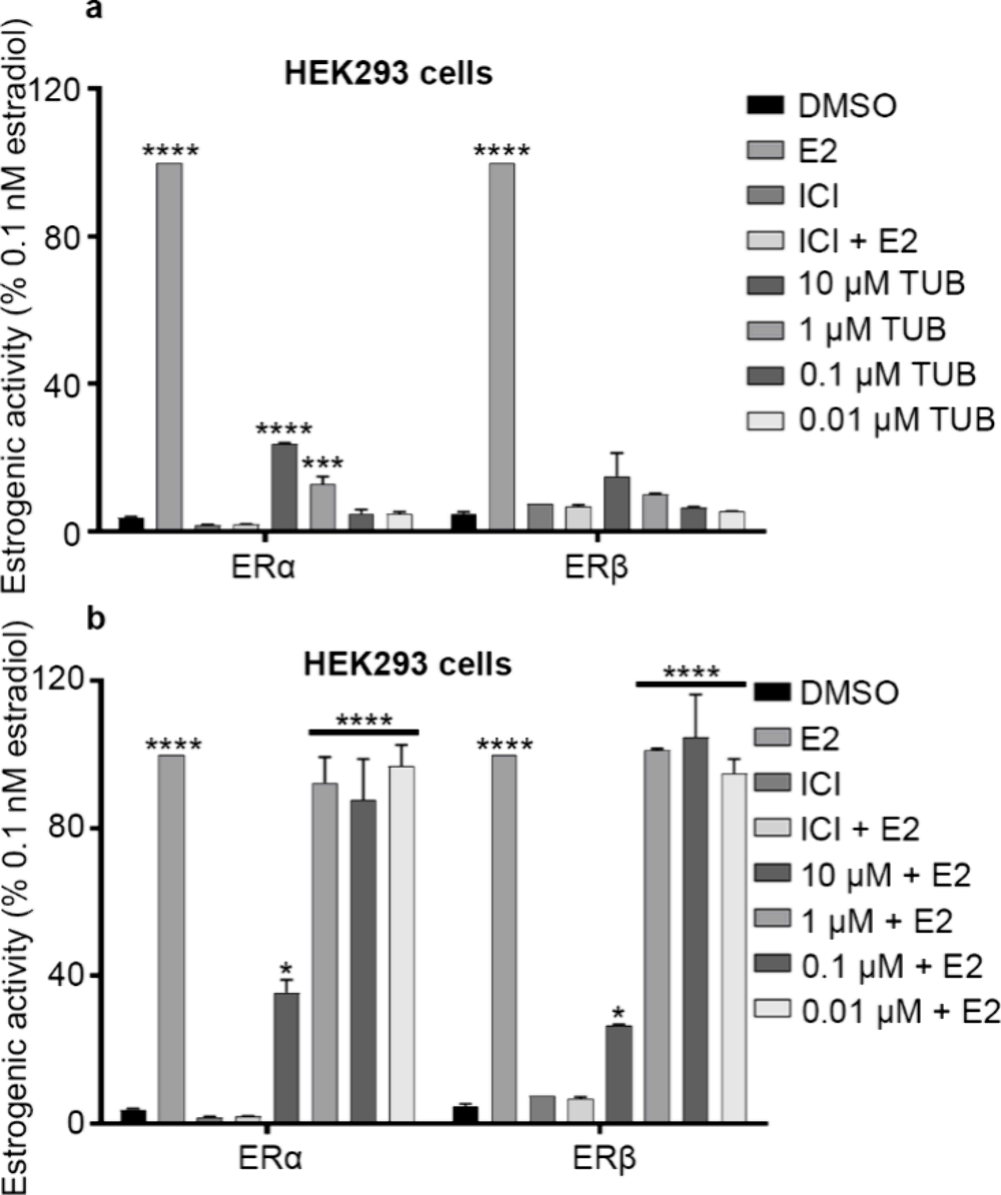
Tuberosin elicits dual estrogenic and antiestrogenic activities in cells with an exogenous ER. HEK293 cells were transfected with 0.2 μg of ER(2)-luc plasmid, 0.2 μg of pcDNA3.1B-ERβ, or 0.2 μg of pcDNA3.1B-ERα plasmids. After a 6 h transfection, cells were treated with DMSO, E2 (0.1 nM), ICI (1 μM), ICI (1 μM) + E2 (0.1 nM), or 0.01, 0.1, 1, or 10 μM tuberosin (TUB) (a) without or (b) with E2. After 18 h, cells were evaluated via a luciferase assay. Data represent mean ± SEM of 3 independent experiments. One-way ANOVA, Dunnett’s multiple comparisons test, compared to DMSO. *p < 0.05, **p < 0.01, ***p < 0.001, and ****p < 0.0001.

To compare the effects of tuberosin in exogenous ER-expressing cells, we analyzed tuberosin-treated ER+ breast cancer cells. In MCF-7 (Figure 3a) and T-47D (Figure 3b) cells, treatment with 10 μM tuberosin, compared to the DMSO control, did not significantly alter estrogenic activity as determined by an ERE-luciferase assay but did increase the activity for both cell lines. Tuberosin treatment (10 μM) in addition to E2 did not significantly decrease the E2-induced estrogenic activity in both cell lines, in contrast to competitive inhibition of ER by tuberosin observed in HEK293-ER cells (Figure 2b). These results suggest that the antagonist effects of tuberosin when combined with E2 is context-dependent. We then assessed the interaction/binding affinity of tuberosin and the ER binding domain.

**Figure 3:**
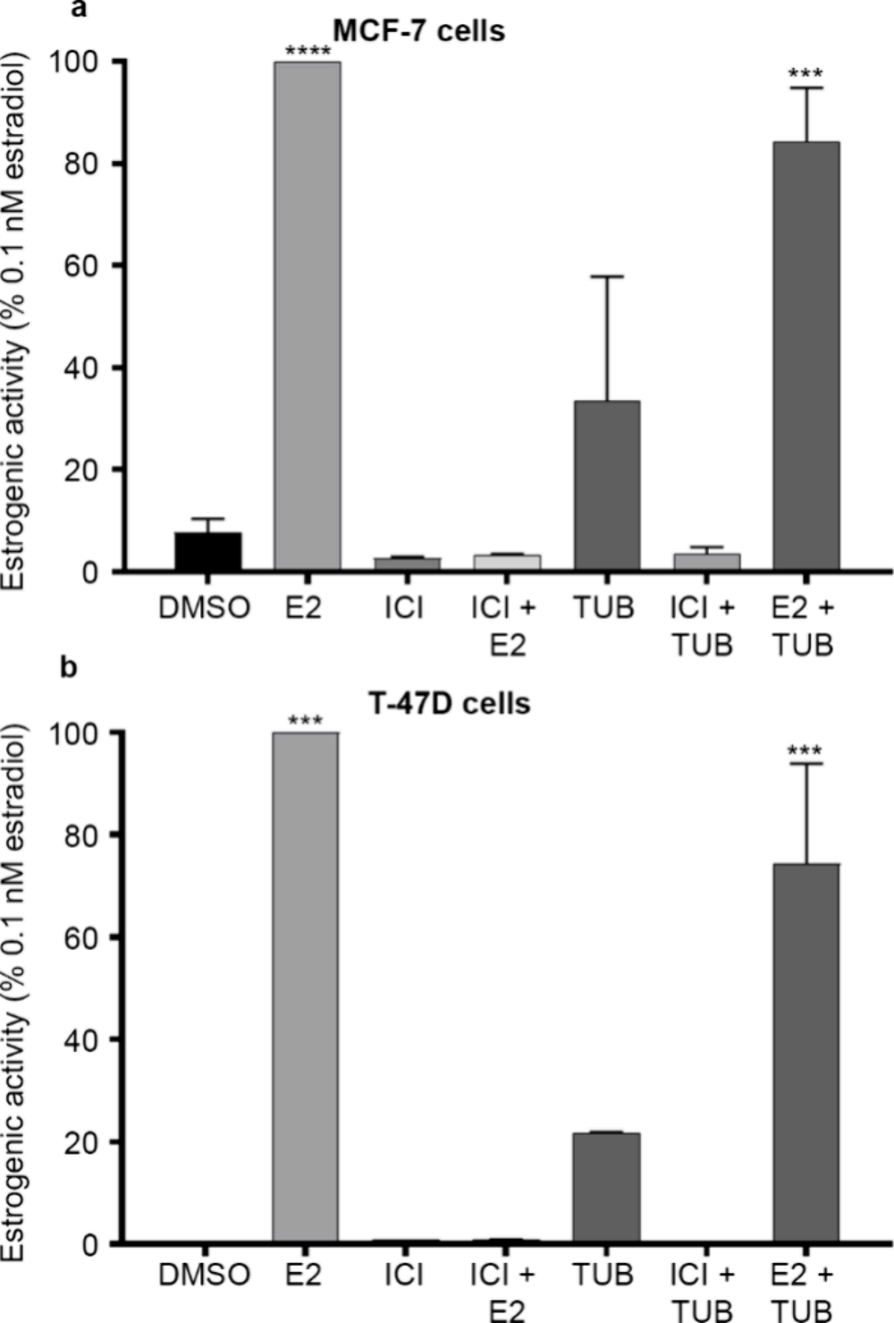
Tuberosin does not act as an ER antagonist in cell lines with endogenous ER. (a) MCF-7 or (b) T-47D cells were transiently transfected with the pGL2-ERE2x-TK-luciferase plasmid. After a 6 h transfection, cells were treated with DMSO, E2 (0.1 nM), ICI (1 μM), ICI + E2, tuberosin (10 μM), or tuberosin + E2. Data represent mean ± SEM of 3 independent experiments. One-way ANOVA, Dunnett’s multiple comparisons test, compared to DMSO. ***p < 0.001, ****p < 0.0001.

### Evaluation of Tuberosin Estrogen Receptor-α Binding Affinity and Molecular Docking Simulations

To establish the binding affinity of tuberosin to the ERα pocket, we used a binding affinity assay to determine the IC_50_ required to produce a > 50% displacement, based on the recorded fluorescence. Figure S5 presents the displacement and concentration responses for tuberosin. The IC_50_ of tuberosin was calculated to be 2,130 nM, which is ∼7,000 times greater than the IC_50_ of the positive control, E2, at 0.283 nM. While higher concentrations of tuberosin are required, compared to E2, tuberosin exhibited a notable affinity for the ERα binding pocket, in accordance with our estrogenic/antiestrogenic activity data. Binding conformation and orientation were determined by molecular modeling techniques.

The active conformation of the binding site that has the estradiol ligand is a hydrophobic cavity created by 12 helices (H1–H12). The helical domain is arranged in three layers, with the binding cavity in the lower half of the domain. The binding cavity is formed by the helices H3, H4, and H12. In the crystal structure, estradiol residing in the binding cavity formed two hydrogen bonds. The phenolic hydroxyl forms a hydrogen bond to the side chain carbonyl of Glu353, the side chain guanidinium group of Arg394, and a H_2_O molecule. The secondary alcohol at the other end of the molecule forms a hydrogen bond to the side chain imidazole nitrogen of His524. Tuberosin generated only two binding modes that had hydrogen bonding interactions in the binding pocket. The binding mode 1 (Figure 4a) with a score of *S* = −4.4583 had the tuberosin molecule in an orientation similar to estradiol, with the phenolic hydroxyl group residing between H3 and H6 making a hydrogen bond with the backbone carbonyl of the residue Leu387 (Figure 4b). The binding mode 2 of tuberosin (Figure 4c) had a binding score of *S* = −3.8060, with the molecule flipped in the binding pocket relative to the first binding mode. In binding mode 2, the phenolic hydroxyl group made a hydrogen bond with the backbone carbonyl group of Gly521 in H11 (Figure 2d).

**Figure 4:**
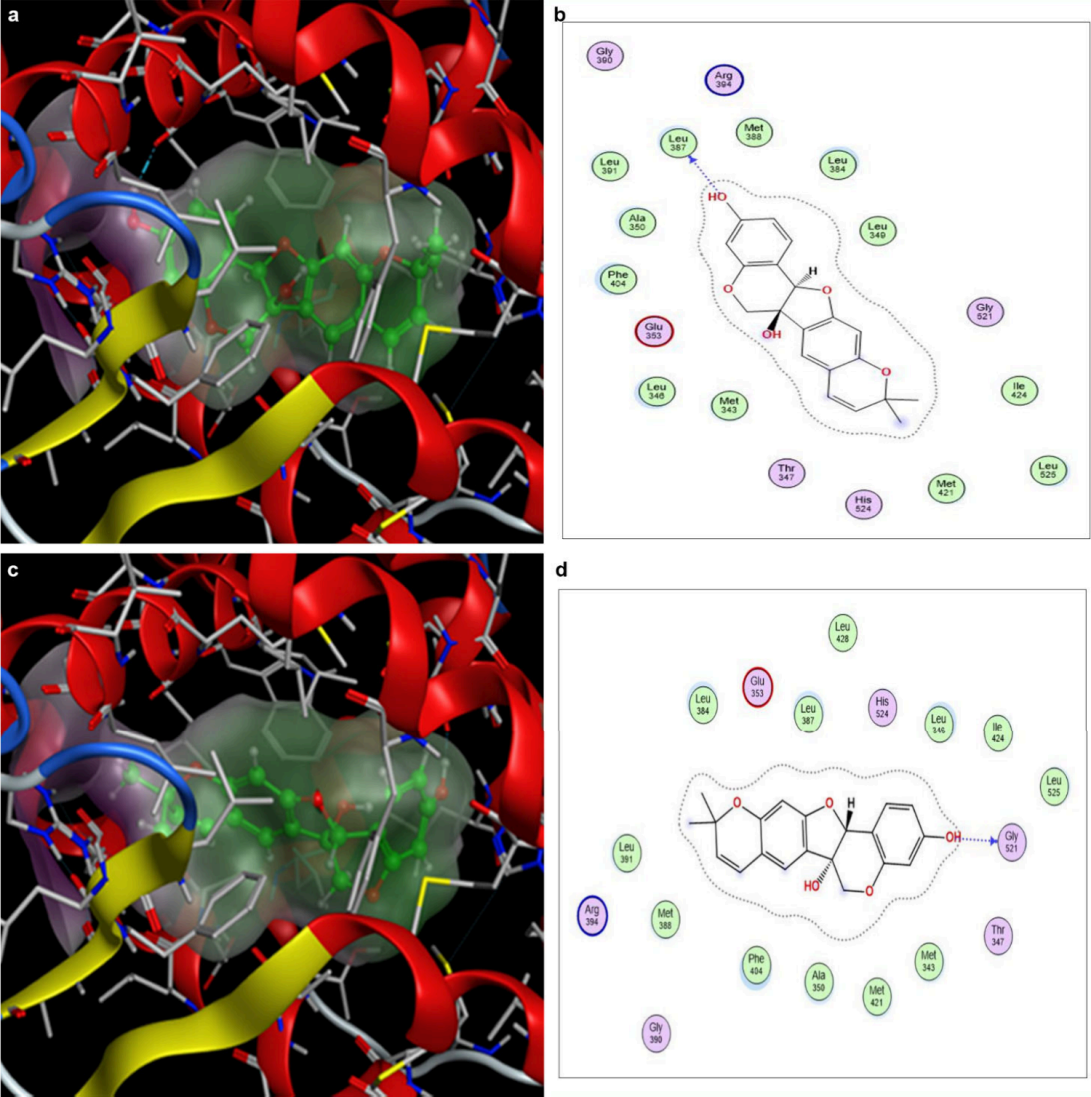
Tuberosin binds to the ERα pocket with two binding modes to elicit estrogenic activity. (a) 3D depiction of binding mode 1 of tuberosin with the phenolic end nestling between H3 and H6. (b) Ligand interaction view of tuberosin depicting the single hydrogen bond in binding mode 1. (c) 3D depiction of binding mode 1 of tuberosin with the phenolic end residing near H11. (d) Ligand interaction view showing the hydrogen bond in binding mode 2.

### Tuberosin Treatment Induces an Estrogen Response Transcriptomic Signature

To determine global transcriptomic effects of tuberosin in ER+ cells, MCF-7 cells were treated for 24 h with tuberosin (10 μM), and RNA sequencing was performed. The early and late estrogen response pathways were the top pathways altered based on genes upregulated following treatment with tuberosin (Figure 5a), further supporting tuberosin as an ER mediator. ER-associated targets induced by tuberosin include PGR, KRT19, TFF1, CXCL12, SERPINA1, SERPINA3, and CCND1, which are all known as estrogen response genes in MCF-7 cells (Figure 5a, b). Additional top pathways included Myc targets and oxidative phosphorylation signaling (Figure 5a). Downregulated genes were associated with cholesterol homeostasis, mitotic spindle, and interferon response. Genes significantly altered by tuberosin treatment include the mTORC1-associated gene RICTOR and cell proliferation mediator Ki67.

**Figure 5:**
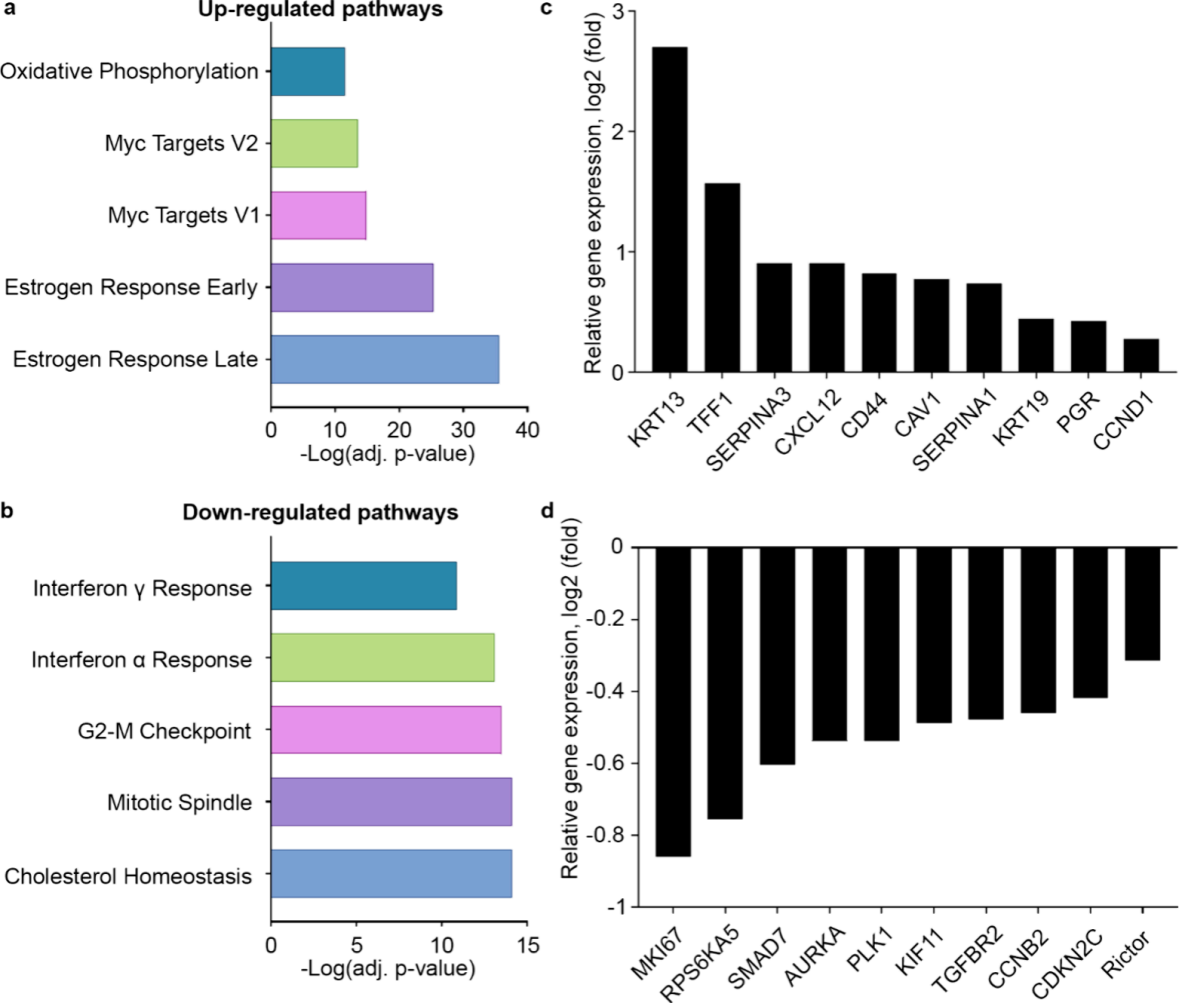
Tuberosin induces the estrogen response gene signature in MCF-7 cells. MCF-7 cells were treated with a 10 μM concentration of tuberosin for 24 h. At end point, total RNA was extracted, and RNA sequencing was performed. Results demonstrate pathway analysis using Enrichr and the MSigDB Hallmark gene set database for top (a) up-regulated and (b) down-regulated pathways. (c) Relative gene expression for estrogen response genes significantly changed in RNA-sequencing of MCF-7 cells treated with tuberosin. (d) Relative gene expression for genes significantly down-regulated in RNA-sequencing of MCF-7 cells treated with tuberosin. Data represent mean ± SEM of 3 independent experiments, and comparisons were to vehicle control (DMSO).

To further elucidate tuberosin-induced changes in ER signaling, the expression of ERα, PGR, and SDF1 was determined by qPCR. ER-mediated gene expression has previously been assessed in the evaluation of novel phytocompounds.^23^ To elucidate the effects of tuberosin, ER+ MCF-7 and T-47D cells were pretreated with ICI (1 μM), followed by either tuberosin (10 μM) or E2 (0.1 nM) treatment. At the end point, gene changes were observed for MCF-7 and T-47D cells treated with tuberosin (Figure 6). There was no observed change in ER in either the MCF-7 or T-47D cell line. SDF1 and PGR expression (*p* < 0.0001) was significantly increased following tuberosin treatment compared to DMSO in both cell lines (Figure 6). ICI alone and in combination with tuberosin or E2 significantly decreased PGR expression in MCF-7 and T-47D cells (*p* < 0.001 and *p* < 0.05, respectively). Given the observed changes in ER-mediated genes, we then assessed effects of tuberosin on protein expression using JESS analysis.

**Figure 6:**
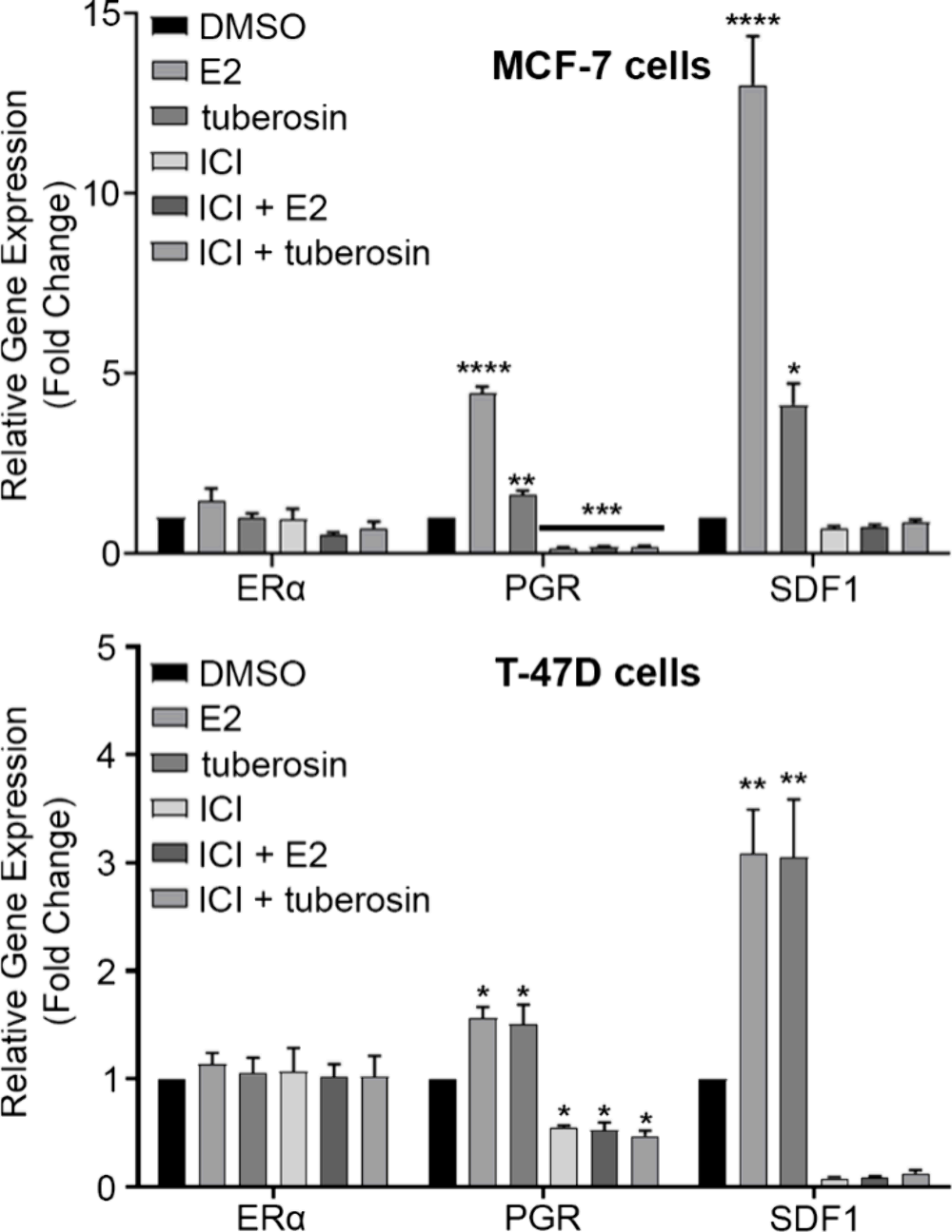
Tuberosin induces estrogen response genes in ER+ cell lines. Cells were grown in 5% DCC stripped media for 48 h. After 48 h, 5 μL of DMSO, E2 (0.1 nM), tuberosin (10 μM), or ICI (1 μM) was added, cells were incubated for 4 h, and then 5 μL of DMSO was added to the E2, DMSO, and tuberosin flasks and 5 μL of E2 or tuberosin was added to the ICI flasks. At end point, cells were collected, total RNA was extracted, and cDNA was synthesized for qRT-PCR. QRT-PCR was performed for ERα and ER response genes, PGR and SDF1, in MCF-7 and T-47D cells. Data were normalized to DMSO control and housekeeping gene (β-actin or RPL13A). Data represent mean ± SEM of 3 independent experiments. One-way ANOVA, Dunnett’s multiple comparisons test, compared to DMSO. *p < 0.05, **p < 0.01, ***p < 0.001, and ****p < 0.0001.

### Total Protein Expression Remains Unchanged with Tuberosin Treatment of MCF-7 Cells

Ligand-dependent ubiquitination and protein degradation was previously shown to be a useful tool in evaluating ligand activity.^24^ Tuberosin treatment in MCF-7 cells did not significantly affect ERα protein expression, while ICI treatment significantly decreased the expression (*p* < 0.0001) compared to DMSO (Figure 7b, Figures S6–8). Based on the changes in estrogenic activity, gene regulation, and the observed regulation of proliferative marker Ki67, in our RNA sequencing data, we next investigated tuberosin effects on cell proliferation.

**Figure 7:**
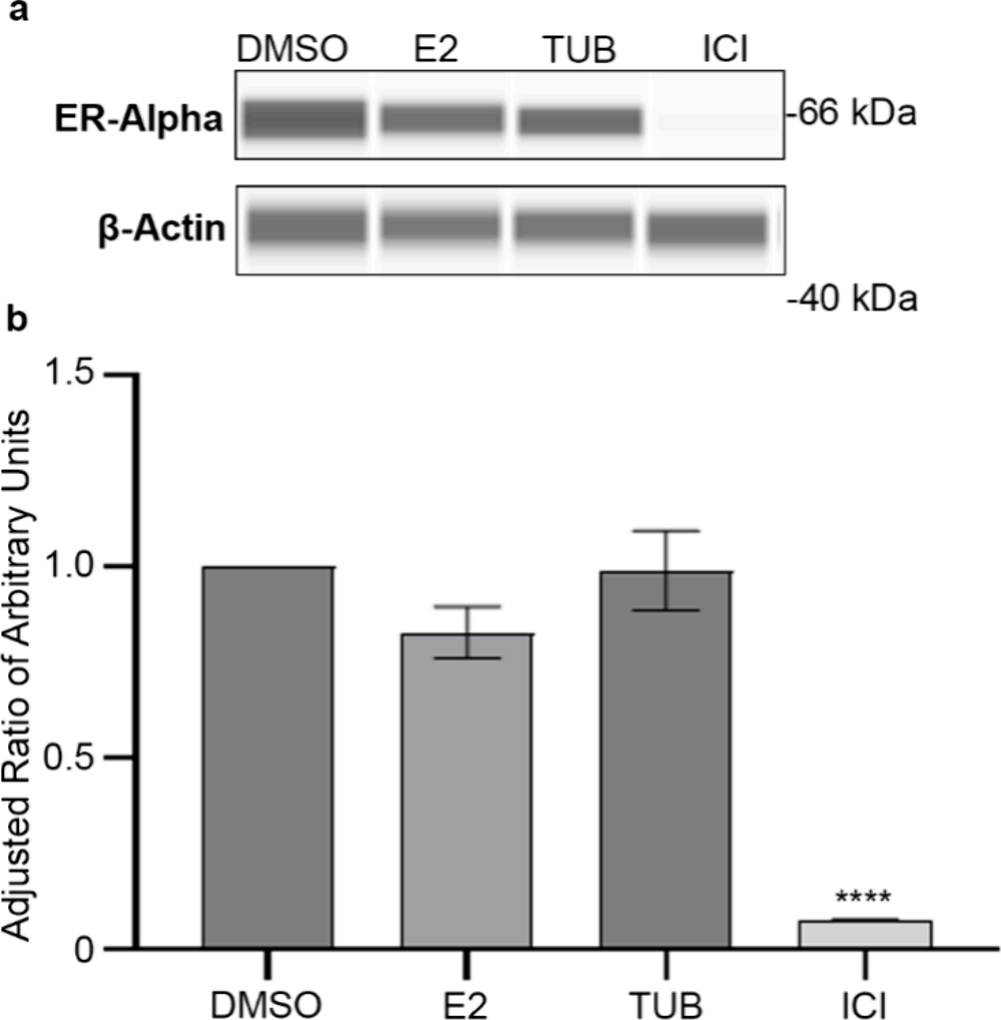
Modulation of ERα protein expression remains unchanged after 30 min of treatment with tuberosin. (**a**) Representative JESS bands for treated MCF-7 cells and (**b**) JESS bands were quantified and plotted to compare changes in expression. Data represents mean ± SEM of 3 independent experiments and data was normalized to DMSO control. One-way ANOVA, Dunnett’s multiple comparisons test, compared to DMSO. ****p < 0.0001.

### Tuberosin Induces Proliferation in a Dose-Dependent Manner

To assess the impact of tuberosin on cell proliferation, increasing concentrations of tuberosin were evaluated on MCF-7 cells using our previously developed protocol.^25^ These studies suggest that tuberosin promoted proliferation in a dose-dependent manner, with treatment of 10 μM tuberosin significantly increasing cell proliferation compared to the DMSO control (Figure 8). Similar to our previous findings, 10 μM tuberosin + 0.1 nM E2 resulted in a nonsignificant reduction in cell proliferation compared to E2 alone. Interestingly, 10 μM tuberosin + 1 μM ICI resulted in much higher cell proliferation (>20%) compared to ICI alone (<1%) but was not significantly different. With tuberosin’s effect on gene and protein expression, the observed proliferation changes, and the regulation of proliferative markers, we next evaluated tuberosin’s effect on vasculogenesis using HUVECs.

**Figure 8:**
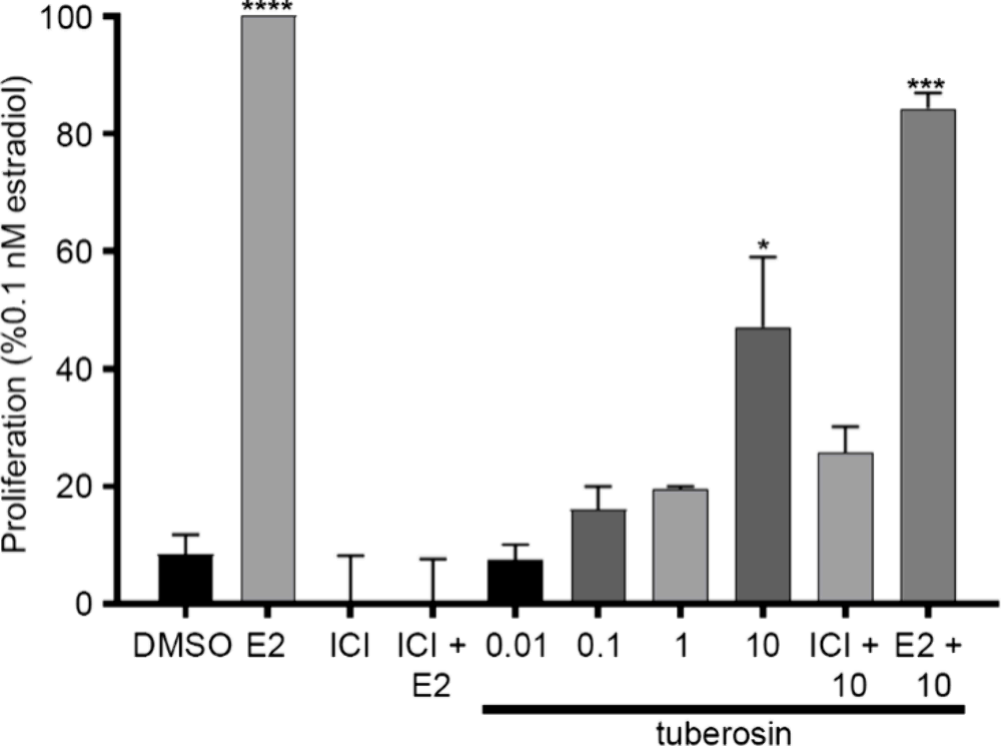
Effects of tuberosin on the Proliferation in MCF-7 cells. Cells were grown for 24 h and then treated with increasing DMSO, E2 (0.1 nM), ICI (1 μM), ICI + E2, or 0.0.1, 0.1, 1, or 10 μM of tuberosin (alone or combined with ICI or E2) for 24 h. Proliferation was evaluated using AlamarBlue and normalized to the positive control (0.1 nM E2). Data represent mean ± SEM of 3 independent experiments. One-way ANOVA, Dunnett’s multiple comparisons test, compared to DMSO. **p < 0.01, ***p < 0.001, and ****p < 0.0001.

### Tuberosin Inhibits Vasculogenesis without Altering Cell Cycle and Density of HUVECs

To evaluate the tissue-scale functional effects of tuberosin on human endothelial cells, bulk tissue vasculogenesis was modeled using a microphysiological system, which has been used previously to examine the effects of phytochemicals.^26^ Here we utilized a custom PDMS device to constrain 3D vascularized tissues composed of female HUVEC and female (XX) human lung fibroblasts (HLF) embedded in fibrin and collagen type I hydrogel. Tuberosin (10 mM) qualitatively disrupted vascular network formation relative to control tissues maintained with standard VEGF-A-containing medium after 7 days of culture (Figures 9a and b). Previous studies have shown, using a similar vasculogenesis assay configuration, that treatment with glyceollin also inhibited microvascular network formation.^26^ Morphometric analysis quantitatively evaluated tuberosin effect on the treated tissues. Tuberosin significantly decreased vessel area fraction (*p* < 0.05), vessel length density (*p* < 0.01), vessel length (*p* < 0.01), and branchpoint count (*p* < 0.05) for treated tissues (Figures 9c–f).

**Figure 9:**
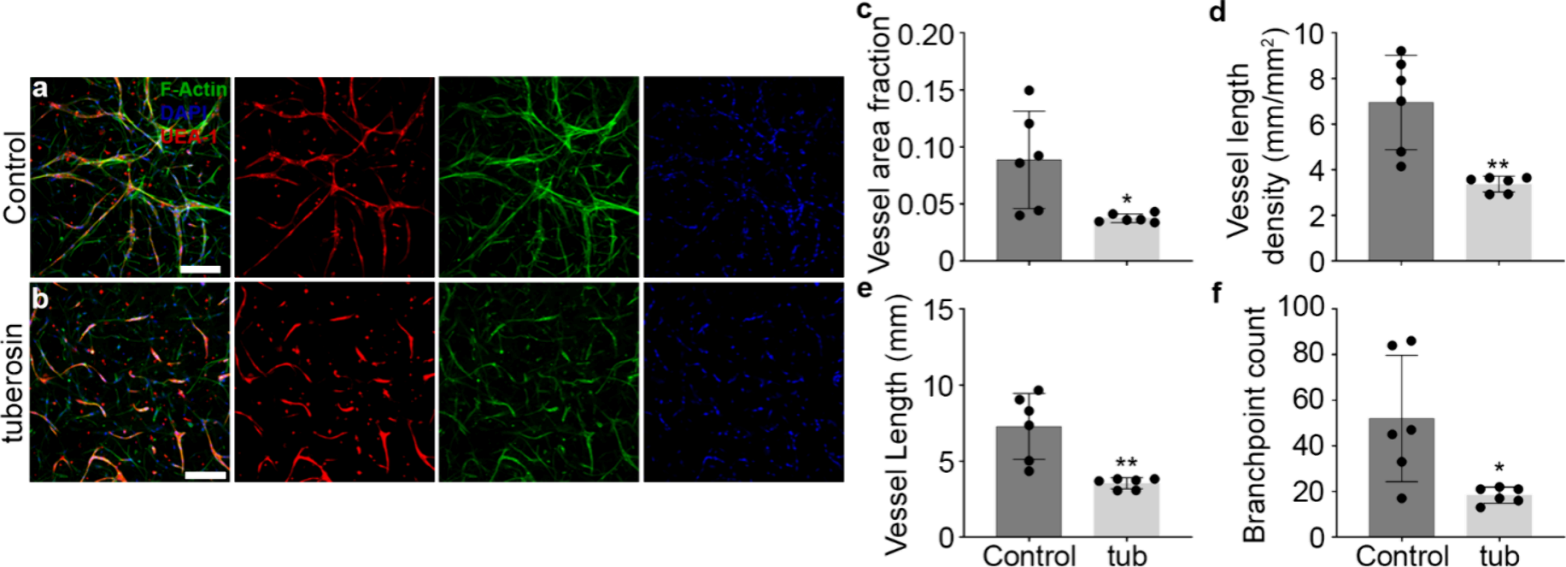
Antivasculogenic effects of tuberosin in engineered bulk tissue vasculogenesis. (a, b) Representative 3D laser scanning confocal microscopy (LSCM) images of the microvascular network in bulk tissue after 7 days in a culture treated with 10 μM tuberosin. Control groups are treated with equal volumes of DMSO. Endothelial cells are labeled with UEA-1 (red). F-Actin in all cells is labeled with phalloidins (green), and fibroblasts are green only. Nuclei of all cells are labeled DAPI (blue). Scale bar: 250 μm. (c–f) Morphometric analysis of resultant vessels after treatment with tuberosin (*n* = 3): (c, d) vessel coverage, (e) size dimensions, and (f) complexity. Statistical analyses were performed using an unpaired *t* test with Welch’s correction. Comparisons are with control groups (*p < 0.05, **p < 0.01, ***p < 0.001, ****p < 0.0001). Error bars represent SEM.

We next evaluated the effects of tuberosin on proliferation of HUVECs in 2D expansion cultures by assessing the cell cycle status using a Ki67 proliferation marker on tuberosin (10 mM) treated cells (Figures S9a and c). The average cell density and Ki67 proliferation index did not change compared to those of control groups (Figure S9b and c). However, there was a qualitative disruption in F-actin organization indicating cellular stress^27^ (Figure S9a).

Taken together, these findings indicate that tuberosin hinders the vascular network assembly and maturation without affecting cellular proliferative capacity. These data suggest that tuberosin may be suppressing the ability of endothelial cells to generate tension needed to migrate rather than exerting a proliferative inhibitory effect.^28^

## Conclusions

The invasive nature of kudzu has contributed to its classification as a weed, as it frequently outcompetes native species, leading to extensive overgrowth. Efforts to control kudzu have proven challenging, with moderate success using both physical and biological agents. In this study, we utilized UVC irradiation and *M. verrucaria* to elicit tuberosin production in kudzu leaves, significantly increasing the tuberosin yield. Our findings demonstrate that estrogenic activity of tuberosin is cell-type-dependent, displaying minimal antagonist or competitive inhibition when combined with E2 in MCF-7 and T-47D cells, while showing dose-dependent agonist activity in HEK293 cells. Additionally, the highest concentration of tuberosin (10 μM) combined with E2 exhibits antagonist or competitive inhibition in HEK293 cells. Tuberosin was shown to modulate ER pathways, alter ER-mediated gene expression, and increase cell proliferation in a dose-dependent manner while maintaining consistent ER-α protein levels. This study highlights the potential of kudzu compounds not only for controlling invasive plant species but also for their valorization in biomedical applications. Our future research will investigate antioxidant properties of tuberosin and explore other potential biomedical applications, such as wound healing.

## Experimental Section

### General Experimental Procedures

A UPLC system (Acquity H-Class, Waters Corp., Milford, MA) with a binary solvent manager, a column manager, and a sample manager were used to identify tuberosin. The sample was separated using a Waters Acquity BEH C-18 column (150 mm × 2.1 mm, 1.7 mm) with a column temperature of 40 °C. The mobile phase consisted of H_2_O containing 0.1% formic acid (A) and MeCN containing 0.1% formic acid (B) with a flow rate of 0.3 mL/min. The gradient elution program was as follows: mobile phase B from 10% to 30% (0–10.00 min), from 30% to 80% (10.00–15.00 min), from 80% to 10% (15.00–17.00 min), and maintained at 10% (17.00–20.00 min). The temperatures of the column and autosampler waswere controlled at 40 and 20 °C, respectively, and the injection volume was 5 μL. A Xevo G2-XS QTOF mass spectrometer (Waters Corp., Milford, MA) equipped with an ESI source was operated in positive ionization mode. The mass spectrometer parameters were as follows: capillary voltage, 2.00 kV; sampling cone, 15 V; extraction cone, 4.0 V; source temperature, 120 °C; desolvation temperature, 300 °C; cone gas flow rate, 50 L/h; and desolvation gas flow rate, 600 L/h. Leucine-enkephalin was used as the lock mass, generating an [M + H]^+^ ion (*m*/*z* 556.2771) to ensure accuracy during the MS analysis. The data was collected in centroid mode by MS^E^ acquisition. The MS^E^ experiment in two scan functions was carried out as follows: function 1 (low energy): mass-scan range 100–600 Da, scan time 0.2 s, interscan time 0.05 s, collision energy 2 eV; function 2 (high energy): mass-scan range 100–600 Da, scan time 0.2 s, interscan time 0.05 s, collision energy ramp 20–30 eV. MassLynx and UNIFI software (Waters Corp., Milford, MA) were used for the postacquisition analysis (Waters Corp., Milford, MA). The nuclear magnetic resonance (NMR) spectra were recorded on a Bruker DRX-500 system (Karlsruhe, Germany) and are presented in Figure S3. The solvent used was deuterated DMSO, which gave a residual signal at 2.50 ppm in ^1^H NMR and 39.50 ppm in ^13^C NMR. The carbon positions are indicated in Figure S4, which uses numbering frequently used for pterocarpans.^31^

Kudzu leaves were obtained from the USDA Agricultural Research Service (Stoneville, MS) and Idadere Farms, LLC (Grand Coteau, LA). HPLC grade MeOH, MeCN, and H_2_O were used as solvents for the study and were purchased from Fisher Scientific (Pittsburgh, PA). A tuberosin standard was obtained from Biorbyt (Durham, NC) and prepared in DMSO at a concentration of 10 mM (stock solution). The final concentration of DMSO was adjusted to less than 0.1% (v/v). Commercially available tuberosin was purchased from Bench Chemicals (Pasadena, CA); 17β-estradiol (E2), fulvestrant (ICI 182, 780), and DMSO were obtained from Sigma-Aldrich (St Louis, MO).

### *M. verrucaria* and UVC Elicitation of Kudzu Leaves

Elicitation with *M. verricaria* was performed as previously described.^5^ Briefly, 2 × 10^7^ of *M. verrucaria* conidia per mL in distilled H_2_O and 2% Silwet L-77 were sprayed on kudzu plants grown in field experiments as described previously.^5^ Treated kudzu leaves were harvested at 15 days after inoculation. Kudzu leaves were treated with UVC irradiation using an Air Science UVC Box (Fort Meyers, Fl) with 3 × 254 nm 60 W bulbs emitting 52.5 W of UVC radiation. The distance between bulbs and leaf samples was 13 cm, and treatments were performed for 14 min, at room temperature. Elicited and nonelicited leaves were placed in plastic trays, covered to avoid dehydration, and incubated for 3 days. Samples were stored at 25 °C, in the dark, for 3 days before being transferred to −70 °C.

Kudzu leaves were lyophilized and ground using a Tekmar A-10 mill (Staufen, Germany). Ground leaf material (1 g) was extracted three times with 5 mL of MeOH for 1 h using ultrasonic treatment (Branson 1510, Danbury, CT). The extracted solvent was filtered through 0.45 μm Nylon filters (Thermo Fisher Scientific, Waltham, MA). Resulting extracts were centrifuged at 10,000g for 20 min (Eppendorf 5415C, Hamburg, Germany), decanted, filtered, concentrated using a Thermo Savant SpeedVac SPD2030 system (Thermo Fisher Scientific, Waltham, MA), and dissolved in DMSO at a concentration of 100 mg/mL.

### Isolation, Identification, And Quantification of Tuberosin from Elicited Kudzu

Tuberosin was isolated from UVC-elicited leaves using semipreparative HPLC techniques developed at the Southern Regional Research Center (ARS, USDA, New Orleans, LA).^29^ Briefly, a Whatman ODS-2 10 mm × 500 mm column was used with a flow rate of 3.0 mL/min and the following solvent system: A = MeCN, B = H_2_O; 5% A for 15 min, then 5% A to 90% A in 40 min, followed by holding at 90% A for 20 min. MeCN was removed from fractions containing tuberosin and lyophilized to remove H_2_O. Tuberosin purity ≥ 98% was achieved based on UPLC analyses.

Identification of tuberosin was achieved by comparing its retention time and UV–vis, MS, and high-energy MS (MS^E^) spectra with those of an analytical standard. The positive ion electrospray mass spectrum of tuberosin isolated from UVC elicited kudzu leaves and the standard is shown in Figure S1. The MS spectrum displays the predominant [M + H – H_2_O]^+^ ion at *m*/*z* 321.1125 and the less abundant ion [M + H]^+^ at *m*/*z* 339.1227. The loss of H_2_O in electro-spray ionization (ESI)-MS spectra has been noted in other pterocarpans such as the glyceollins and occurs with loss of the 6a-hydroxyl group.^30^ The ESI-MS/MS spectra for tuberosin from stressed kudzu leaves and the standard are shown in Figure S2.

Quantification of tuberosin was performed using UPLC analyses on a Waters Acquity H-Class instrument with a PDA detector. A Waters Acquity BEH C-18 column (150 mm × 2.1 mm, 1.7 mm) was used at a flow rate of 0.3 mL/min with a 5 mL injection volume. Peak areas of tuberosin were quantified at 309 nm. A linear calibration curve (*R* ^2^ > 0.99) for tuberosin was prepared between a range of 4–200 μg/mL. Reported data are the mean ± SEM of three independent experiments, and concentration was reported as μg/g on a dry weight basis.

### Cell Culture

All cells were cultured in 150 cm^2^ culture flasks in Dulbecco’s modified Eagle’s medium (DMEM; Invitrogen, Carlsbad, CA) supplemented with 10% fetal bovine serum (FBS, Life Technologies, Inc., Gaithersburg, MD), basic minimum MEM essential (50×, Invitrogen, Carlsbad, CA) and MEM nonessential amino acids (100×, Invitrogen, Carlsbad, CA), sodium pyruvate (100×, Invitrogen, Carlsbad, CA), antimycotic-antibiotic (10,000 U/mL penicillin G sodium, 10,000 μg/mL streptomycin sulfate, 25 μg/mL amphotericin B as fungizone, Invitrogen, Carlsbad, CA), and human recombinant insulin (4 mg/mL, Invitrogen, Carlsbad, CA). For assays requiring cells to be cultured in stripped media, the following were used: phenol red-free DMEM (Gibco, Billings, MT), 5% dextran-coated charcoal-treated FBS (DCC, HyClone, Logan, UT), penicillin streptomycin (Gibco, Billings, MT), basic minimum MEM essential and MEM nonessential amino acids, sodium pyruvate, and Glutamax (100×, Gibco, Billings, MT). Cells were maintained in a humidified tissue culture incubator maintained at 37 °C and 5% CO_2_.

Human umbilical vein endothelial cells (HUVEC, ATCC, Manassas, VA) were maintained in vascular cell basal medium supplemented with Endothelial Cell Growth Kit-VEGF (ATCC, Manassas, VA) and 2% FBS (ATCC, Manassas, VA). Human lung fibroblasts (HLF, ATCC, Manassas, VA) were maintained in fibroblast basal medium supplemented with Fibroblast Growth Kit-Low Serum (ATCC, Manassas, VA) and 2% FBS. All culture media contained 1% antibiotic-antimycotic (Corning, Corning, NY). Cells were maintained in a humidified tissue culture incubator maintained at 37 °C and 5% CO_2_. Cells were harvested at 80%–90% confluency, and experiments were performed using cells between passages 3 and 6.

### ERE-Luciferase Assay

MCF-7 and T-47D cells were cultured in stripped medium for 48 h prior to plating. Cells were plated (5 × 10^5^ cells/well) in 24-well plates (Corning, Corning, NY) and allowed to adhere overnight. The next day, cells were transfected with 0.1 μg of ER(2)-luc plasmid (Panomics, Fremont, CA) using Effectene transfection reagent (Qiagen, Hilden, Germany), following the manufacturer’s protocol. After 6 h, the cells were treated with tuberosin and incubated at 37 °C. DMSO, E2 (0.1 nM), or ICI (1 μM) was used as the control. Combinations of extracts and ICI or E2 were also evaluated. Media were removed after 18 h, replaced with 200 μL/well of 1× lysis buffer (Promega, Madison, WI), incubated for 15 min at room temperature, and then pelleted by centrifugation at 15000*g* for 5 min. Cell extracts were normalized for protein concentration using Bio-Rad Reagent following the supplied protocol (Bio-Rad Laboratories, Hercules, CA). Luciferase activity was determined using 1× Luciferase Assay Substrate (Promega, Madison, WI) in a Lumat LB 9510 luminometer (Bertholdt, Bad Wildbad, Germany). Reported data for MCF-7 cells represent the mean ± SEM of three independent experiments and two independent experiments for T-47D cells.

HEK 293 cells were cultured and plated as stated above. After 24 h, cells were transfected with 0.05 μg of ER(2)-luc plasmid (Panomics, Santa Clara, CA) and either 0.05 μg of pcDNA3.1B-ERb or 0.05 μg of pcDNA3.1B-ERa plasmids using Effectene transfection reagent (Qiagen, Valencia, CA), according to the manufacturer’s protocol. After 6 h, cells were treated with tuberosin and incubated at 37 °C. DMSO, E2 (0.1 nM), or ICI (1 μM) was used as the control. Combinations of tuberosin and ICI or E2 were also evaluated. The next day, cells were lysed with 150 μL of the M-Per mammalian extraction reagent (Pierce, Rockford, IL). 100 μL of cell extract was assayed using the Bright-glo luciferase assay substrate (Promega, San Luis Obispo, CA) and determined in a Berthold AutoLumat Plus luminometer. Reported data are the mean ± SEM of two independent experiments.

### ERα Binding Assay

Tuberosin was assessed for ERα binding affinity using the ThermoFisher LanthaScreen binding assay (Waltham, MA). Following the manufacturer’s protocol, all reagents were prepared and brought to room temperature before use. Briefly, stock solutions were prepared for tuberosin, DMSO (no ligand), and E2 (control ligand) and serially diluted using the provided buffers. Then, 10 μL of each solution was transferred to the provided plate. Finally, 5 μL of Fluormone ES2 Green tracer and 5 μL of an ER Alpha-LBD (GST) and terbium anti-GST antibody solution were added to the wells. The plate was placed in a shaker incubator, protected from light, and incubated at room temperature. Using an Agilent Biotek Cytation5 (Santa Clara, Ca), fluorescent readings were taken at 495/520 nm, and the TR-FRET ratio was calculated by dividing the emissions at 520 nm by 495 nm. IC_50_ values (tuberosin and E2) were calculated by graphing the emission ratio vs the log [ligand]. Reported data are the mean ± SEM of two independent experiments.

### Molecular Docking and Modeling

The 3D-structure of the C-terminal ligand-binding domain (LBD) of wild type estrogen receptor-α (WT ERα) in complex with estradiol (3ERE.pdb) was downloaded from the RCSB Protein Data Bank (RCSB PDB) Web site. The Molecular Operating Environment (MOE) modeling software from the ChemComp group was used for the docking studies. The 3D structure of tuberosin was built using the “Builder: modules of MOE. Required hydrogens were added to the structure, assigned partial using the MMFF94 force field, and energy minimized to a gradient of 0.1 RMS kcal/mol/AΛ2. Tuberosin was subjected to conformational search application in the LowModeMD, which is a short molecular dynamics simulation using velocities with little kinetic energy in the high-frequency vibrational modes due to its generality and efficiency. Only one conformational structure was generated for tuberosin. The crystal structure of WT ERa-LBD in complex with estradiol (3ERE.pdb) was stripped of H_2_O molecules and prepared for docking using the “QuickPrep” module of MOE to correct structural issues and protonation states. Docking studies were performed using the “Dock” module of the MOE software. The alpha triangle matcher placement method was used for the placement of ligands in the binding pocket. Poses were generated by aligning ligand triplets of atoms on triplets of alpha spheres in a more systematic way than that in the alpha triangle method. The London dG scoring function that estimates the free energy of binding of the ligand from a given pose was initially used. A rescoring of binding pose was done using the GBVI/WSA Δ*G*, that is, a force field-based scoring function, which estimates the free energy of binding of the ligand from a given pose. This rescoring was initially performed with rigid receptor constraints, followed by an induced fit mode where the side chains of the protein residues were free to move. The output had top 5 poses with the top “S” scores (the final binding score). The results were evaluated through visual inspection of the docked complexes. The criteria for visual inspection were that the ligand should reside in the ATP binding pocket of the protein close to the hinge region. The ligand pose that satisfied the initial visual inspection, and with the lowest “S” score, was taken as final binding pose.

### RNA Sequencing

MCF-7 cells were grown in stripped media for 48 h prior to treatment with DMSO or 10 μM tuberosin for 24 h. At the end point, total RNA was extracted, quantified, and assessed for integrity using a Bioanalyzer (Agilent Technologies, Santa Clara, CA). The total RNA was extracted using a Direct-zol RNA purification kit (Zymo research, Irvine, CA). The mRNA was then purified from total RNA using oligo­(dT)-attached magnetic beads and fragmented into small pieces. The fragmented mRNA was synthesized into first-strand cDNA using random primers. The second strand of cDNA was synthesized with dUTP instead of dTTP. The cDNA thus synthesized was subjected to end-repair and 3′-adenylation. Subsequently, adaptors were ligated to the ends of the 3′-adenylated cDNA fragments. The U-labeled second-strand template was digested with Uracil-DNA-Glycosylase (UDG). PCR amplification was performed, and sequencing on the DNBSEQ (DNBSEQ Technology) platform was performed. After reads filtering, clean reads were mapped to reference the human genome using HISAT, and gene expression values were identified by TPM and detected by RSEM. Results represent gene changes as Log2 fold change. Unbiased pathway analysis was performed through Enrichr Pathways and the MSigDB Hallmark gene set. Genes included in the analysis were all significantly changed genes with adjusted p-values.^32^ The data discussed in this publication have been deposited in NCBI’s Gene Expression Omnibus^33^ and are accessible through GEO Series accession number GSE277758.

### Quantitative PCR

MCF-7 and T-47D cells were seeded at ∼5,000 cell/cm^2^ (25 cm^2^ flasks, VWR, Radnor, PA) and grown to ∼70% confluency. The medium was then aspirated, and the cells were washed 3× with PBS. 5 mL of stripped media was added, and the cells were incubated for 48 h. After 48 h, 5 μL of DMSO, E2 (100 nM), tuberosin (10 mM), or ICI (100 μM) was added to the corresponding flasks, and the flasks were incubated for 4 h. After 4 h, 5 μL of DMSO was added to E2, DMSO, and tuberosin flasks, and 5 μL of E2 (100 nM) or tuberosin (10 mM) was added to the ICI flasks. After 24 h, cell pellets were collected, and total RNA was extracted (RNeasy Mini Kit, Qiagen, Hilden, Germany) and reverse transcribed to cDNA (iScript cDNA SuperMix, BioRad, Hercules, CA) following the manufacturer protocol. Quantitative PCR was performed (BioRad CFX Connect Real-Time System, v4.006; BioRad, Hercules, CA and BioRad IQ SYBR green Supermix, BioRad, Hercules, CA) as per the manufacturer’s protocol, and expression was calculated using the ΔΔ­(Ct) method and reference gene β-actin. Primer sequences are presented in Supplementary Table S1. Samples were normalized to vehicle control gene expression (DMSO) and designated as 1. Reported data are the mean ± SEM of three independent experiments.

### Cell Proliferation Assay

MCF-7 cell proliferation was assessed using previously published methods.^25,34^ Briefly, cells were cultured in stripped media for 7 days prior to plating. Cells were then plated (4.5 × 10^3^ cells/well) in 96-well plates (Corning, Corning, NY) and allowed 24 h for adherence. After 24 h, cells were treated with DMSO, E2 (0.1 nM), ICI (1 μM), or tuberosin (0.01, 0.1, 1, or 10 μM). Combinations of tuberosin and ICI or E2 were also evaluated. Cells were redosed on day 4, and cell proliferation measured on day 7. At end point, Alamar Blue dye (Thermo Fisher Scientific, Waltham, MA) was added to the medium (10 μL/well) and incubated for 3 h at 37 °C with 5% CO_2_. Fluorescence was evaluated at 560/595 nm by using a HTS7000 Series Bio Assay Reader (PerkinElmer, Boston, MA). Reported data are the mean ± SEM of two independent experiments.

### Capillary Western Blot (JESS) Analysis

MCF-7 cells were seeded (26,000 cells/cm^2^) in 6-well plates (Corning, Corning, NY) and grown until ∼70% confluency. Once confluent, the media was aspirated, and the cells were washed with PBS three times and grown in stripped media for 48 h. After 48 h, cells were treated with tuberosin (10 μM), positive control E2 (0.1 nM), ICI (1 μM), or a vehicle control (DMSO) for 30 min. After 30 min, cells were washed with 1 mL of cold PBS and agitated using a cell scraper (VWR, Radnor, PA). A lysing solution was prepared by adding a protease and phosphatase inhibitor cocktail (100×, Thermo Fisher Scientific, Waltham, MA) at 1:100 dilution with the mammalian protein extraction reagent lysis buffer (Thermo Fisher Scientific, Waltham, MA). A 100 μL portion of the solution was then added to each well, agitated to ensure lysing, and then transferred to 1.5 mL Eppendorf tubes (Sigma-Aldrich, St. Louis, MO). Tubes were then briefly vortexed, incubated on ice for 8 min, and centrifuged at 14,000 rpm for 10 min at 4 °C. The supernatant was transferred to a new tube. Total protein was used and diluted following the manufacturer’s protocol.

Capillary Western analyses were performed using the ProteinSimple Jess System using the Wes/Jess separation module (12–230 kDa) (Protein Simple, San Jose, CA) that contains capillary cartridges and prefilled microplates with split running buffer, wash buffer, and sample buffer. The module also has EZ Standard Pack 1 with 5× fluorescent master mix, biotinylated ladder, and DTT. The antirabbit detection Master Kit includes antirabbit secondary antibody, antibody diluent, streptavidin-HRP, luminol-S, peroxide, and wash buffer. All were purchased from Protein Simple and used following the manufacturers protocol. Antimouse secondary antibody was also purchased from Protein Simple. Briefly, 0.1× sample buffer was used to dilute MCF-7 lysates at 1:2. Then, 1 part of the 5× fluorescent master mix (which included the 5× fluorescent standard, 5× sample buffer, and 200 mM DTT) was mixed with 4 parts of the diluted samples and heated for 5 min at 95 °C to denature the protein. Since the molecular weight ladder is only present on the first capillary and each capillary is independent, the system control proteins in the fluorescent master mix function as a “ruler” to standardize the distance for each capillary.^35^ Following the denaturation process, the primary antibodies (ERα, phospho-ERα S118, β-actin 1:1000 dilution), secondary antibodies conjugated with HRP, blocking reagent, and chemiluminescent substrate were added to the appropriate wells in 12–230 kDa separation plates. Antibodies used were specific for ERα (Millipore Sigma, Burlington, MA) and phospho-ERα S118 (Cell Signaling, Danvers, MA). For every experiment, molecular weight standards were supplied via a biotinylated ladder. After the assay plate was loaded, the fully automated capillary system employed the separation electrophoresis and immunodetection. A CCD camera in Jess collects the data. The Compass for Simple Western program (ProteinSimple, San Jose, CA) was used to analyze the data. Areas under the peaks in Jess were noted to present the relative amount of protein. Reported data are the mean ± SEM of three independent biological replicates.

### Tuberosin Mediated Vasculogenesis

#### Ki67 Immunofluorescent Staining and Quantification

HUVECs were seeded at 10,000 cells per well in 48-well tissue culture plates and cultured for 24 h. The cells are treated with DMSO or tuberosin (10 μM) in the endothelial cell growth medium for an additional 24 h. 2D plated HUVECs were fixed in 4% paraformaldehyde (PFA, Polysciences) for 15–20 min and washed with PBS. The samples were blocked and permeabilized with 1% bovine serum albumin (Sigma-Aldrich, St. Louis, MO) and 0.1% triton X-100 (Sigma) for 15 min and incubated with primary antibodies against Ki76 index marker (Abcam, Cambridge, United Kingdom) for 2 h at room temperature at a 1:100 concentration in 0.1% BSA in PBS. After washing, secondary antibody goat antirabbit IgG (Alexafluor 594, Abcam, Cambridge, United Kingdom) was dissolved in 0.1% BSA solution at dilution of 1:500 along with DAPI (Hoechst 33342, Thermo Fisher, Scientific, Waltham, MA) and phalloidin (Alexa488, Invitrogen, Thermo Fisher Scientific) at 1:500 dilution at room temperature in the dark. Stained tissues were washed with PBS and stored at 4 °C, protected from light until imaging. To quantify the Ki67 index, cell nuclei of spheroids (stained with Hoechst 33 342) were segmented into discrete regions of interest (ROI) using a watershed transformation.^36^ The mean fluorescence within each nucleus-ROI was computed, and a minimum threshold was set to classify cells as positive or negative for Ki67. Ki67 index was reported as the fraction of positively fluorescing cells in each image. Cell density is quantified using DAPI positive nuclei. All image analysis was completed in MATLAB (R2023b).

#### Stereolithography Printing, Postprocessing, and PDMS Casting

Device molds were drafted as 3D drawings using SolidWorks (SolidWorks Corp., Waltham, MA) or Fusion 360 (Autodesk Inc., San Francisco, CA). Molds were created in a top-down view and printed using a Form 3B SLA Printer (Formlabs Inc., Somerville, MA). Final designs were exported as “.STL” files to Formlab’s PreForm software. Formlab’s PreForm software was used to prepare 3D drawing files for printing. The part file was oriented so that the mold base was parallel with the printer’s build platform. Print supports were autogenerated within PreForm with a 0.65 mm touchpoint size and a 1.30 support density. All molds were printed using Formlab’s proprietary “Clear” SLA resins.

Completed prints were washed in i-PrOH according to Formlabs protocols for the resins used and then washed in i-PrOH for 20 min in the FormWash (Formlabs Inc., Somerville, MA). Postwash, molds were dried until i-PrOH was completely evaporated. Molds were then dried and cured under UV light (FormCure, Formlabs Inc., Somerville, MA) at 60 °C for 15 min. Warping was caused by distortion of the printing process, and the curing process was corrected by first baking molds at 130 °C for 2 h. For the last 30 min of bake time, 2 stainless steel jeweler’s blocks were added to the oven to heat. Molds were removed and placed between two jeweler’s blocks with or without clamping. Flatness of the parts was assessed visually relative to a straight edge. We used a commercial painting airbrush (Badger Airbrush Co., Bellwood, IL) to coat molds with lacquer thinner (Klean-Strip, Memphis, TN). Parts were dried for 15 min while preparing an automotive clear coat (Sherman Williams, Cleveland, OH) mixed at a 1:4 ratio of hardener to clear coat. The mixture was airbrushed from approximately 8 in. away using a continuous back and forth motion and then a continuous up and down motion until a thin layer of clear coat was visible. Four layers were applied with the mold rotated 90° between applications. Coated molds were dried for 6 h before silanization according to standard protocols for soft lithography.^37^

Polydimethylsiloxane (PDMS) (Sylgard 184, Ellsworth Chemical, Germantown, WI) was mixed at a 1:10 ratio of PDMS curing agent to PDMS elastomer by weight and degassed. PDMS soft lithography was carried out according to standard protocols used for silicon master molds.^38^ To produce PDMS molds with two flat surfaces, we applied a cleaned 2 × 3 in glass slide to the top of the mold to sandwich the uncured PDMS, carefully avoiding bubble formation. A jeweler’s block was placed on top of the slide to ensure a tight seal, and filled molds were baked at 60 °C for at least 4 h.

#### Vasculogenesis Assay

Devices were exposed to UV light in a cell culture hood for 20 min. The surfaces of the chambers of the constrained device that house bulk 3D tissues were functionalized for extracellular matrix (ECM) hydrogel anchorage using a modified version of the polydopamine (PDA, Sigma-Aldrich, St. Louis, MO) coating method as previously described by Park et al.^39^ Briefly, a 5 mg/mL dopamine solution was prepared by mixing dopamine hydrochloride (Sigma-Aldrich, St. Louis, MO) with 10 mM Tris-hydrochloride (Sigma-Aldrich, St. Louis, MO). Dopamine solution was sterile filtered and pipetted into the inner well of the constrained device. Devices were incubated for 2 h at room temperature under ultraviolet irradiation, aspirated, and washed with ultrapure H_2_O. PDA-treated devices are used within 1 week of coating. Biological sex female HLF and HUVEC (2 × 10^6^ cells/mL each) were admixed in 2.2 mg/mL collagen I (Corning, Corning, NY), 5 mg/mL fibrinogen (Sigma-Aldrich, St. Louis, MO), and 1 U/mL thrombin (Sigma-Aldrich, St. Louis, MO). This cell-inoculated hydrogel precursor was injected into the inner well of the constrained device and incubated for 15 min. The outer chambers were filled with endothelial cell growth medium (ATCC) supplemented with 25 μg/mL aprotinin (EMD Millipore, Burlington, MA) and 2% FBS and replenished every 2 days. Control groups were treated with DMSO, and tuberosin groups were treated at 10 μM, both dissolved in the media starting from the seeding day and replenished every 2 days. Bulk vasculogenesis tissues were cultured for a total of 6 days, with the initial seeding described as day 0.

#### Immunofluorescent Staining in Bulk Vasculogenesis Tissues

Bulk tissues in devices were stained using adaptations of a previously reported protocol.^40^ Briefly, tissues were fixed by loading 4% PFA in the outer chambers of the device and incubating them for 1 h at room temperature. Devices were washed with PBS and stored at 4 °C prior to staining. Tissues were stained using 4 μL/mL DAPI to label nuclei, 4 μL/mL phalloidins to label actin in all cells, and 10 μL/mL DyLight594-conjugated Ulex Europeas agglutinin I (UEA-1, Vector Laboratories, Newark, CA) to specifically label microvascular networks.^41^ The staining cocktail was prepared in 1× PBS with 0.2% Triton-X-100 and 1% BSA. The tissues were incubated with the staining cocktail solution on a rocker for 1 h at room temperature prior to washing with PBS. Stained tissues were loaded with PBS and stored at 4 °C, protected from light until imaging.

#### Imaging and Analysis

Stained tissues were fixed in a position on a glass slide and imaged on an inverted Nikon C2 laser scanning confocal microscope (LSCM) equipped with a Nikon DS-FI3 camera. A large composite containing a 2 × 2 and 3 × 3 stitched image was acquired using a 10× magnification objective lens from each sample of bulk vasculogenesis and 2D Ki67 index proliferation experiments, respectively. For bulk vasculogenesis tissues, 5 evenly spaced z-slices of the stitched images were acquired. Max intensity projections of Z-stacks from vasculogenesis samples were exported as TIFFs for image analysis. All vascular morphometric analysis was completed in MATLAB (vR2021b, MathWorks, Natick, MA). Vascular network images were smoothed using an edge preserving filter with a Gaussian kernel, and a threshold was applied to remove the remaining low-intensity noise. We used a pretrained deep neural network to denoise each image, and adaptive histogram equalization was used to standardize contrast across the image set. We then segmented preprocessed images and quantified morphometric parameters using an open-source automated segmentation tool.^42^

#### Statistical Analysis

Statistical analyses were performed using GraphPad Prism (version 10.0.2, GraphPad Software, San Diego, CA). One-way ANOVA with multiple comparisons (Dunnett’s) or *t* tests were performed to determine the significance of recorded values indicated in the figure legend. *p*-Values of <0.05 were considered significant. Each value is presented as the mean ± standard error of the mean (SEM).

## Supplementary Material



## Notes

Additional experimental data including qRT-PCR primer sequences, MS and MS-MS specra for UVC-elicited kudzu leaves compared to a tuberosin standard, tuberosin molecular structure with marked carbon positions corresponding to NMR, tuberosin ERα binding affinity, estrogen receptor response late genes identified by RNA-sequencing, complete JESS blots for MCF-7 treated cells, and HUVEC morphology and proliferation changes due to tuberosin treatment (PDF)
