# Supplementary material for: Modulation of Estrogen Receptor Activity by the Phytoalexin Tuberosin Produced from Elicited Kudzu (Pueraria lobata)
Source: J Nat Prod. 2025 Jun 20;88(7):1568–80. doi: 10.1021/acs.jnatprod.5c00192 (PMC12305667; doi:10.1021/acs.jnatprod.5c00192)
Supplement: Supplementary file 1 [file np5c00192_si_001.pdf]

Supporting Information

Modulation of Estrogen Receptor Activity by the Phytoalexin Tuberosin, Produced from Elicited Kudzu (*Pueraria lobata*).

Jorge A. Belgodere[1,2,3,#], Jack R. Elliott[1,#], Megan C. Benz[1], G. Wills Kpeli[4], Steven Elliott[1], Isaac J. Ponder[2], Geoffroy S. Pema[1], Peng Ma[5], Sophie R. Dietrich[1], Thomas Cheng[1], Khoa Nguyen[1], Syreeta L. Tilghman[6], Binghao Zou[7], Muralidharan Anbalagan[3,7], Brian G. Rowan[3,7], Robert H. Newman[8], Mark Mondrinos[3,4], Jayalakshmi Sridhar[5], Thomas E. Wiese[5], Simak Ali[9], Van T. Hoang[1,3], Bridgette M. Collins-Burow[1,3], Elizabeth C. Martin[1,3], Hamed K. Abbas[10], Stephen M. Boué[11], Matthew E. Burow[1,3*]*

[1]Tulane Department of Medicine, Section of Hematology & Medical Oncology, Tulane University Health Science Center, New Orleans, LA 70112

[2]Department of Biological and Agricultural Engineering, Louisiana State University and Agricultural Center, Baton Rouge, Louisiana 70803

[3]Tulane Cancer Center, Tulane University, New Orleans, LA 70112

[4]Department of Biomedical Engineering, Tulane University, New Orleans, LA 70112

[5]Xavier University School of Pharmacy, Xavier University, New Orleans, LA 70125

[6]Pharmaceutical Sciences Division, College of Pharmacy and Pharmaceutical Sciences, Florida A&M University, Tallahassee, FL 32307

[7]Department of Structural and Cellular Biology, Tulane University School of Medicine, New Orleans, LA 70112

[8]Department of Biology, North Carolina A&T State University, Greensboro, NC 27411

[9]Department of Surgery and Cancer, Imperial College London Hammersmith Hospital Campus London UK

[10]U. S. Department of Agriculture, Agricultural Research Service, Southeast Area, Stoneville, MS 38776

[11]U. S. Department of Agriculture, Agricultural Research Service, Southern Regional Research Center, New Orleans, LA 70179

[#]Co-first authors

* mburow@tulane.edu; Tel.: (504) 988-6688

* steve.boue@usda.gov; Tel.: (504) 286-4346

Table of contents

Table S1. Quantitative PCR primer sequences. 4
Figure S1. Electrospray high resolution MS spectrum of tuberosin 5
Figure S2. Electrospray high resolution MS-MS Spectrum of tuberosin 6
Figure S3. Tuberosin proton NMR 7
Figure S4. Tuberosin structure 7
Figure S5. Binding affinity of tuberosin to the ERα pocket 8
Table S2. Estrogen receptor response late genes 8
Figure S6. β-Actin JESS blot 12
Figure S7. ER-α JESS blot 13
Figure S8. p-ER-α JESS blot 14
Figure S9. Effects of Tuberosin on Ki67 proliferation index of 2D plated HUVECs 15

Table S1. Quantitative PCR primer sequences.

Gene	Forward	Reverse
Β-actin	AGATGAGTATGCCTGCCGTG	ACATGTCTCGATCCCACTTAAC
RPL13a	GAGGCCCCTACCACTTCC	AACACCTTGAGACGGTCCAG
ERα	GGCATGGTGGAGATCTTCGAA	CCTCTCCCTGCAGATTCATCA
PGR	TACCCGCCCTATCTCAACTACC	TGCTTCATCCCCACAGATTAAACA
SDF1	ACACTCCAAACTGTGCCCTTCA	CCACGTCTTTGCCCTTTCATC

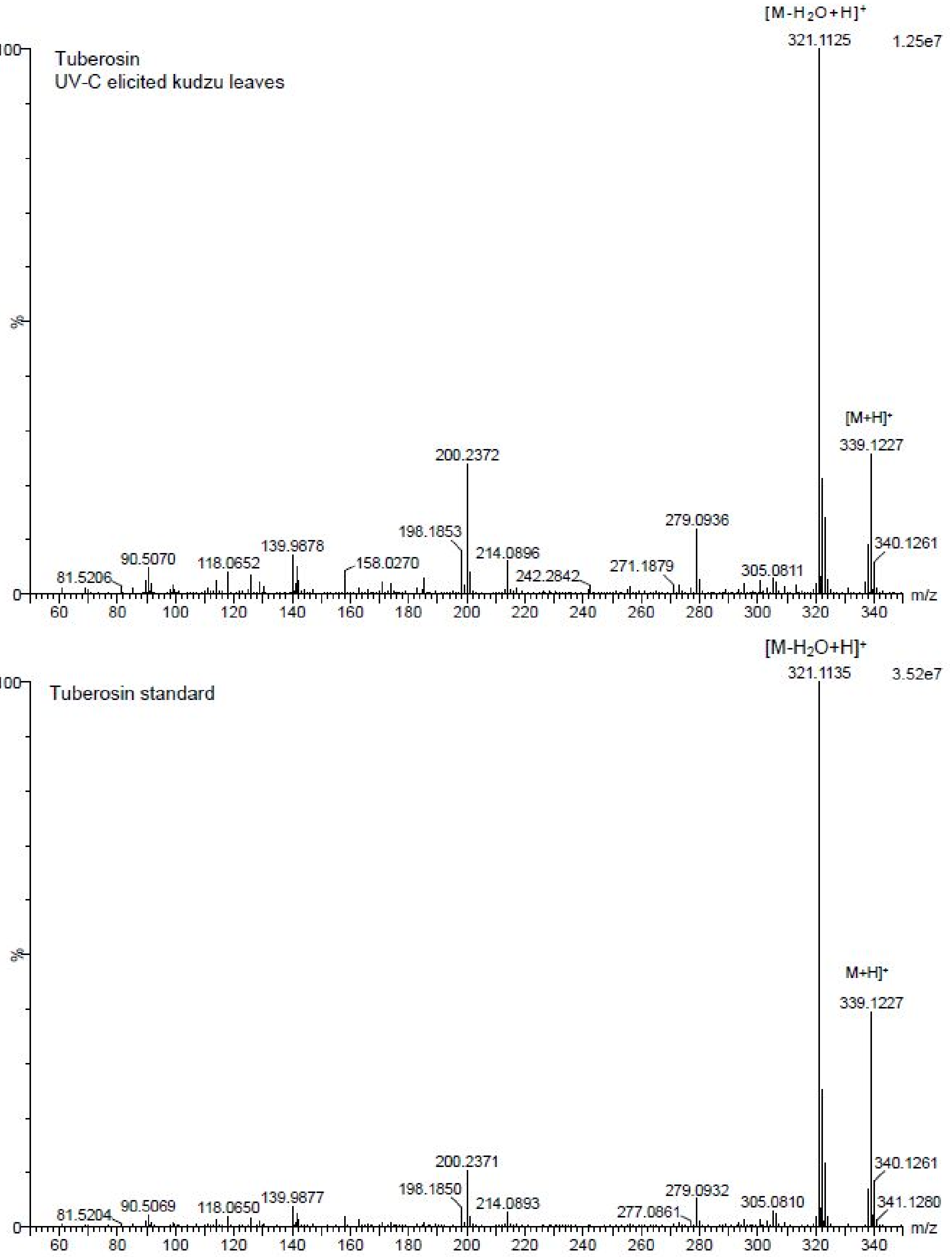

Figure S1. Electrospray high resolution MS spectrum of tuberosin from (top) UVC-elicited kudzu leaves and (bottom) standard.

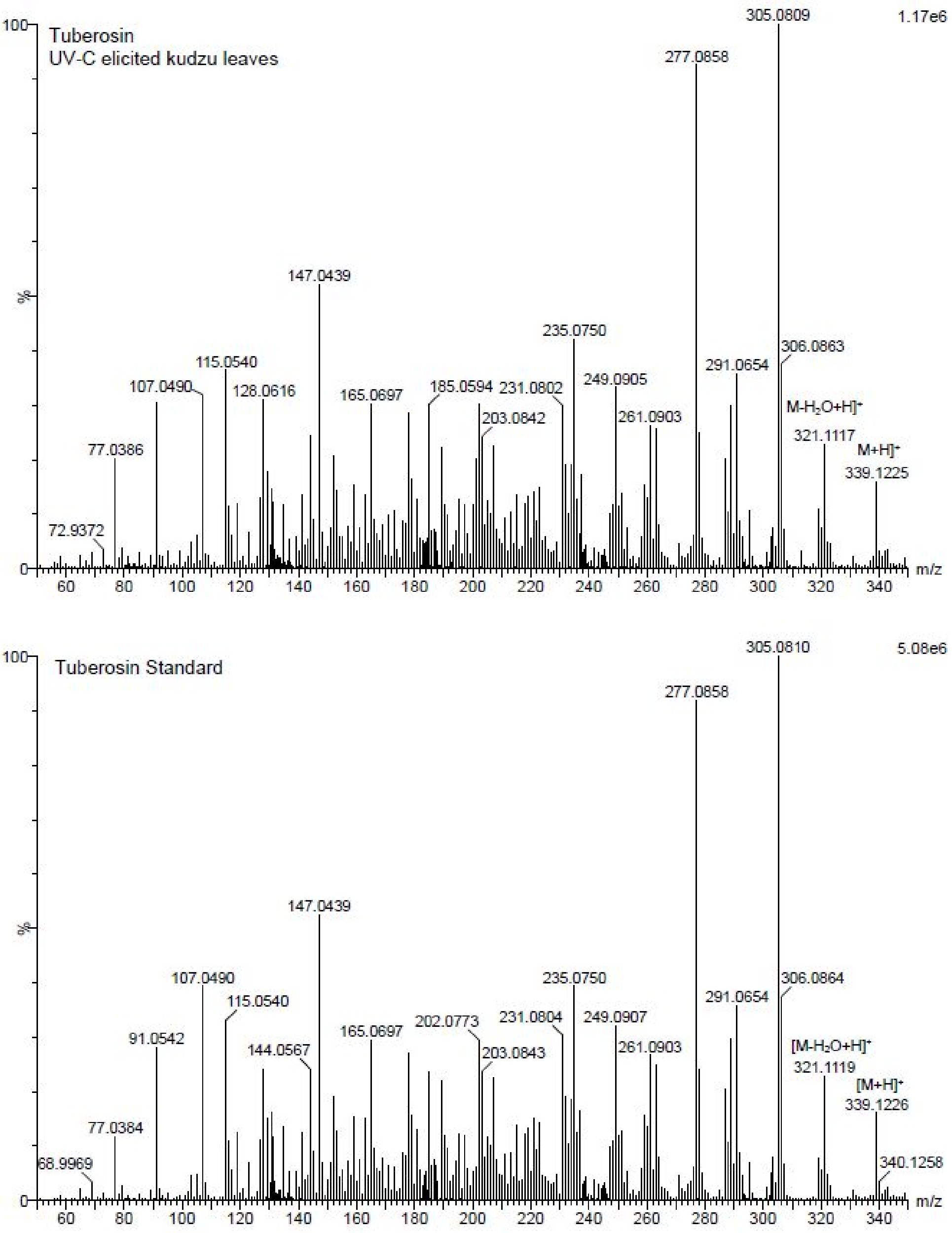

Figure S2. Electrospray high resolution MS-MS Spectrum of tuberosin from (top) UVC-elicited kudzu leaves and (bottom) standard.

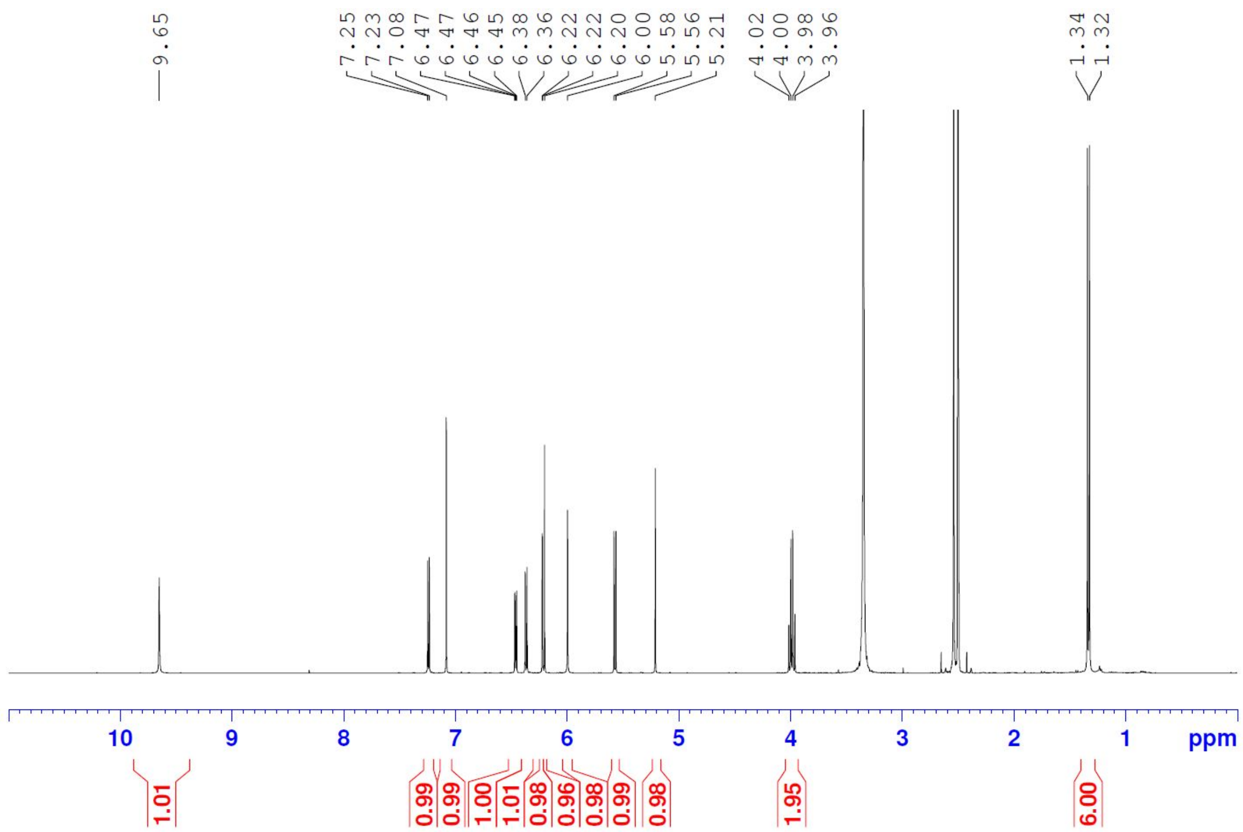

Figure S3. Tuberosin proton NMR spectrum. Residual solvent peaks in NMR spectrum: quintet at 2.50 ppm is from DMSO-d6, singlet at 2.54 ppm is from DMSO, and the singlet at 3.35 ppm is from water.

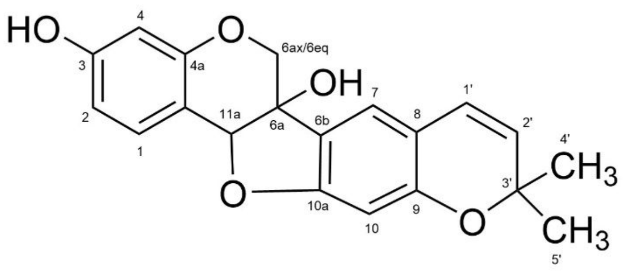

Figure S4. Tuberosin structure with numbered carbon positions for NMR.

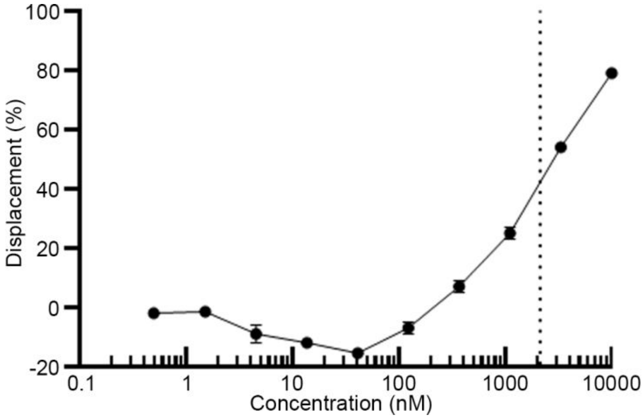

Figure S5. Binding affinity of tuberosin to the ERα pocket requires greater than 1000 nM concentrations to initiate displacement. E2 was used as positive control, with an IC50 of 0.283 nM. Dotted line indicates calculated IC50 value, 2,130 nM. Data represents mean ± SEM of 2 independent experiments.

Table S2. Estrogen receptor response late genes identified from RNA-sequencing.

Estrogen Response Late	log2 (Tuberosin / DMSO)	Qvalue (Tuberosin / DMSO)
KRT13	2.699115341	5.37209E-17
PLAC1	2.355265908	2.55817E-12
CCN5	1.745187149	6.98934E-20
AREG	1.705393717	2.86468E-15
TFF1	1.569438119	5.78261E-47
XBP1	1.409648362	2.63798E-12
FABP5	1.386248563	0.036737815
SEMA3B	1.311805778	3.50842E-17
IGSF1	1.259053886	2.0083E-06
ISG20	1.2002028	6.22163E-09

PTGES	1.115696823	0.03035622
BLVRB	1.067108246	2.67148E-09
ASCL1	0.904368748	0.037293844
SERPINA3	0.901372309	1.15418E-05
CXCL12	0.901075337	8.08955E-07
PDZK1	0.856638165	0.006104335
ALDH3B1	0.845401303	0.00071691
EGR3	0.834953164	2.9854E-05
TFF3	0.834445343	5.86433E-06
LAMC2	0.828953375	5.54881E-05
TMPRSS3	0.828088083	0.023101169
CYP26B1	0.826083626	0.002193635
CD44	0.818852529	4.23961E-14
TPD52L1	0.813447285	5.82672E-11
SERPINA5	0.808425035	0.015171167
CAV1	0.770242947	1.3758E-05
FRK	0.755250626	0.035715175
BATF	0.754614067	0.005958426
SLC7A5	0.750346602	0.00017211
TST	0.744372509	0.000248912
SERPINA1	0.73302816	0.018549642
ETFB	0.703791969	7.45367E-06
PCP4	0.701391123	0.017865665
IGFBP4	0.659731636	6.69223E-07

HSPB8	0.642861237	5.24029E-07
HPRT1	0.641749502	4.80645E-06
DHRS2	0.627209195	0.012111655
ASS1	0.59777466	0.000115483
NAB2	0.58320393	0.000363417
DCXR	0.582653747	0.038540918
DYNLT3	0.576239337	1.24072E-05
MDK	0.566313589	0.009389081
CPE	0.558515246	0.000248456
NXT1	0.550026187	0.013742539
PPIF	0.538347061	5.31752E-06
GJB3	0.538175695	0.005958426
PKP3	0.530177503	0.014609494
OPN3	0.492777428	0.001425101
PRSS23	0.488381641	0.001181444
SLC27A2	0.478909629	0.004224577
XRCC3	0.477542417	0.032808272
KLK11	0.467861136	0.001113219
HSPA4L	0.455059726	0.025226322
KRT19	0.444329305	0.01445742
RAPGEFL1	0.438100013	0.011157118
PGR	0.425579393	0.025318394
ARL3	0.417120958	0.026138622
BAG1	0.404249579	0.018105311

MYB	0.396258183	0.024090027
COX6C	0.384137456	0.046697662
AGR2	0.381307631	0.000865143
KLF4	0.361434683	0.026672352
MOCS2	0.357752408	0.020723454
OLFM1	0.357573628	0.017111758
RAB31	0.337875539	0.014885463
TRIM29	0.320294558	0.029674067
CCND1	0.273485768	0.011473834
CA2	0.25804346	0.035477194

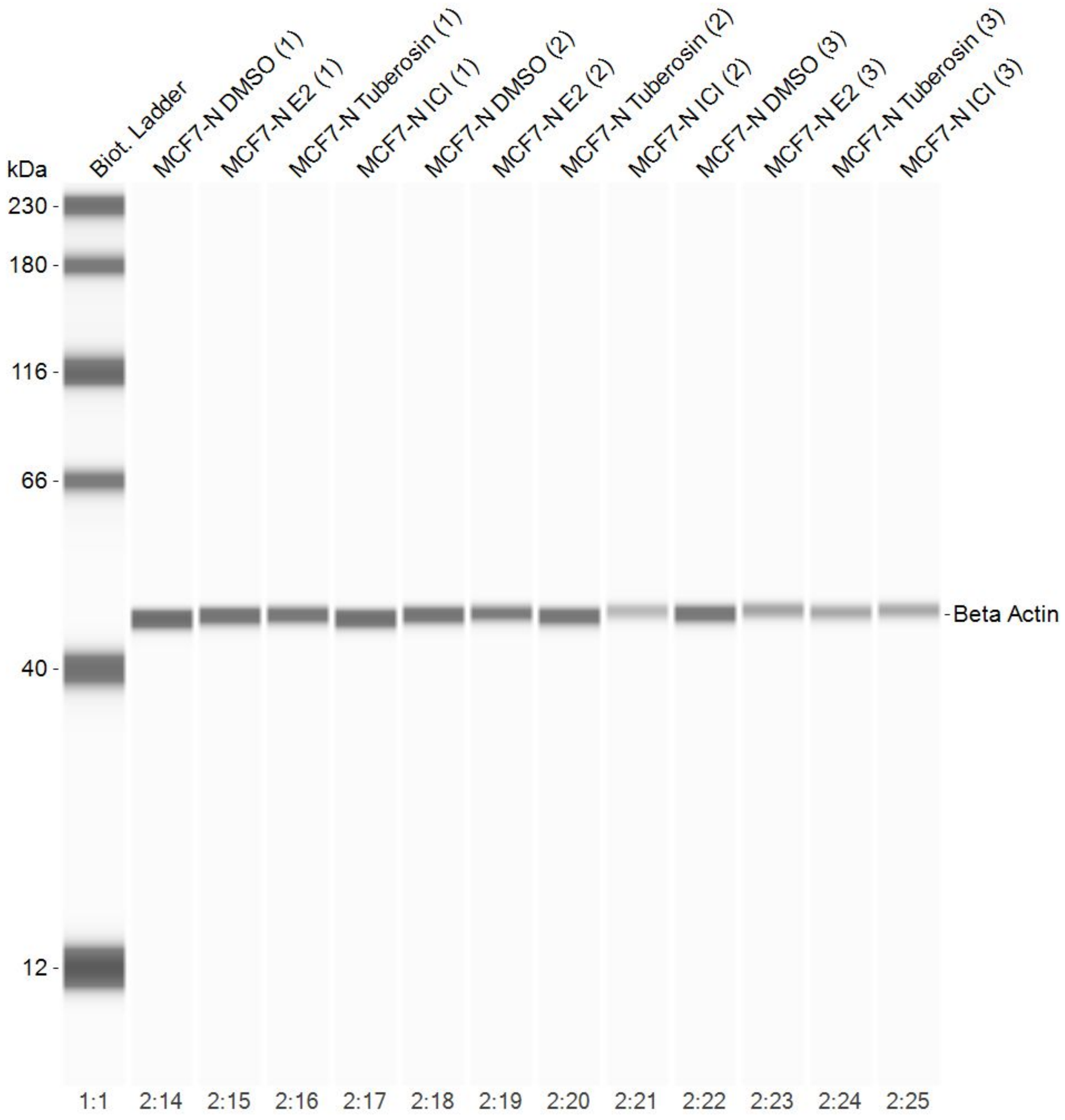

Figure S6. β-Actin JESS blot

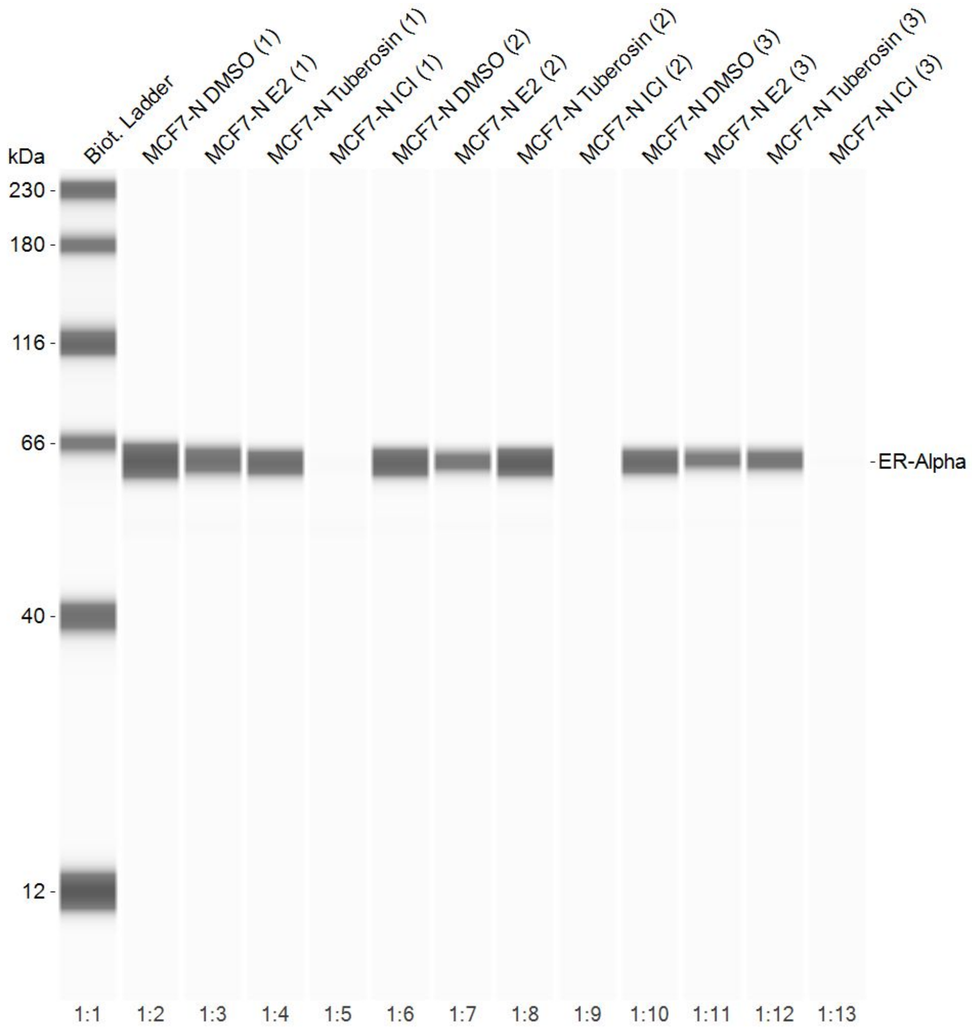

Figure S7. ER-α JESS blot

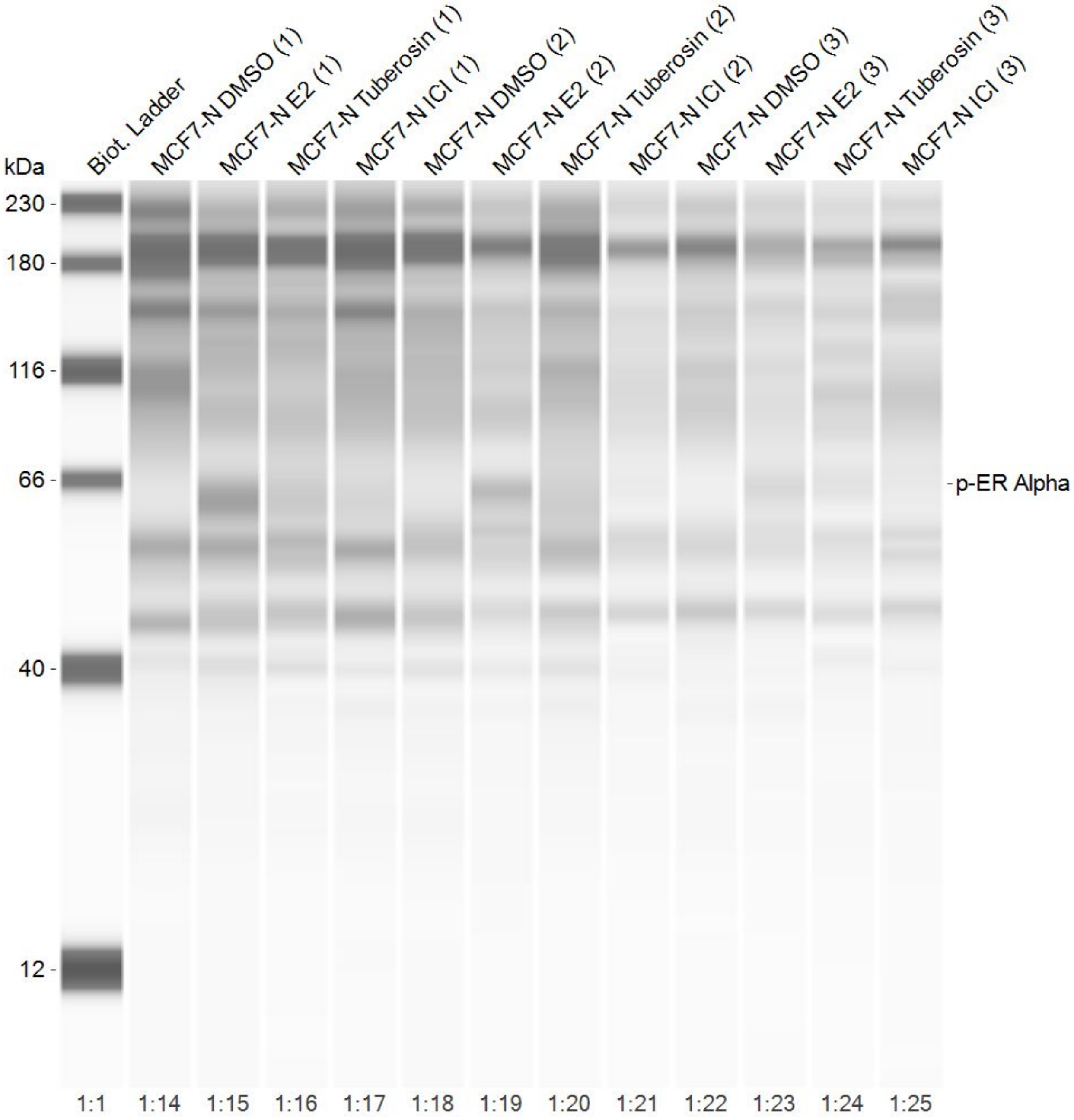

Figure S8. p-ER-α JESS blot

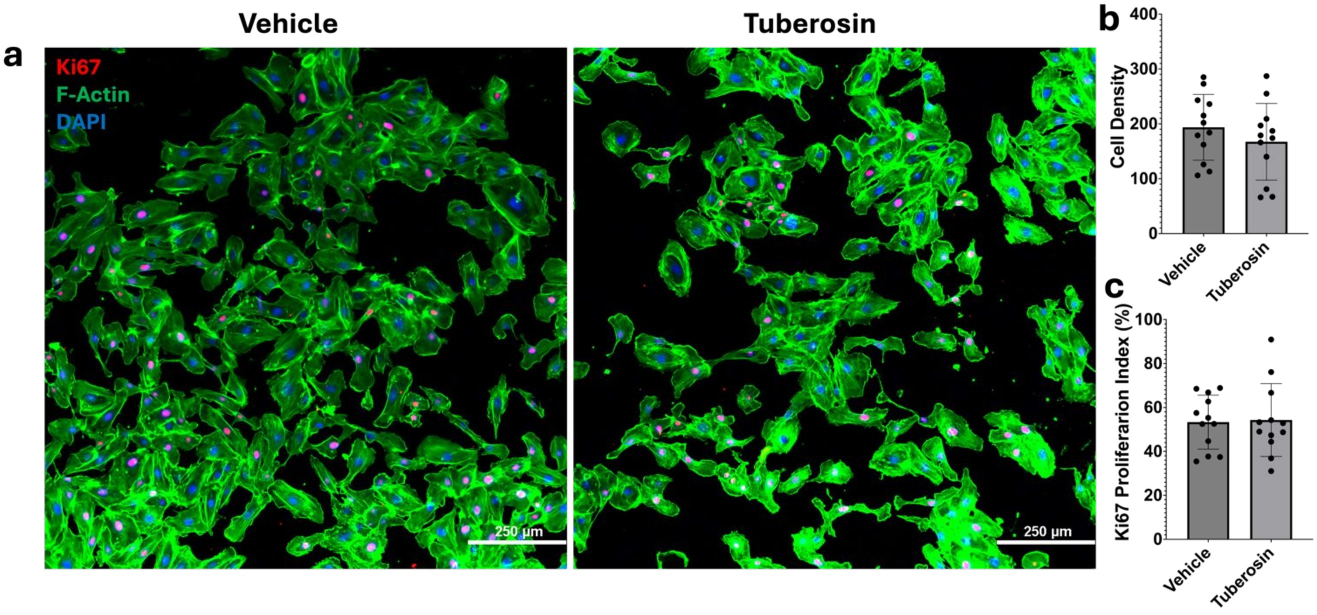

Figure S9. Effects of Tuberosin on Ki67 proliferation index of 2D plated HUVECs. (**a**) Representative laser scanning confocal microscopy (LSCM) images of endothelial cells cultured in 48-well tissue culture plates treated with 10 µM tuberosin. Control groups (vehicle) are treated with equal volumes of DMSO. Ki67 is labelled red. F-Actin in all cells is labelled with phalloidins (green). Nuclei of all cells are labelled DAPI (blue). Scale bar = 250µm. b-c: average cell density (**b**) and mean percentage ki67 proliferation index (c) (n=3). Statistical analyses were performed using an unpaired T-test. (*p<0.05, **p<0.01, ***p<0.001, ****p<0.0001). Error bars represent SEM.
